# The experience of recurring ambivalence and its relation to effortful problem-focused coping

**DOI:** 10.1038/s41598-026-35032-4

**Published:** 2026-01-16

**Authors:** Shiva Pauer, Bastiaan T. Rutjens, Frenk van Harreveld

**Affiliations:** 1 Department of Social Psychology, University of Amsterdam, Amsterdam, The Netherlands; 2 Department of Social Psychology, Helmut Schmidt University, Postfach 700822, 22008 Hamburg, Germany; 3 National Institute for Public Health and the Environment (RIVM), Bilthoven, The Netherlands

**Keywords:** Ambivalence, Coping, Conflict, Decision-making, Self-regulation, Temporal dynamics, Psychology, Human behaviour

## Abstract

Ambivalent attitudes are a pervasive part of people’s lives, yet research has primarily examined isolated instances of ambivalence without considering its persistence across time. The present research addresses this temporal dimension of ambivalence and how it shapes coping efforts. We propose that frequently recurring ambivalence motivates individuals to engage in more effortful coping because they (a) appraise the recurrence as aversive and (b) seek to prevent future reexperiences of ambivalence-induced discomfort. Study 1 revealed variability in how frequently people experience ambivalence across various personal topics, attributable to attitude importance and individual differences. As hypothesized, perceptions of recurrence amplified the correlation between felt ambivalence and effortful problem-focused coping, but without a role of dispositional future-oriented thinking. Study 2 further substantiated this interaction by employing a quasi-experimental design that varied felt ambivalence and recurrence across personal topics, suggesting a role of negative appraisals of recurring ambivalence in its motivational effect on coping. Study 3 employed a decision-making task and manipulated ambivalence and expectations of its future recurrence, yielding no significant interaction effect on effortful information seeking. Taken together, recurring ambivalence is a widespread and often unpleasant experience, and our studies (*N* = 1,672) provide tentative support for the notion that the discomfort of reexperiencing ambivalence (but not heightened expectations of future recurrence) increases the motivation to engage in more effortful resolution attempts. A temporal perspective offers novel implications for understanding the nature of ambivalence and its consequences.

**Supplementary Information:**

The online version contains supplementary material available at 10.1038/s41598-026-35032-4.

Ambivalence is an inherent part of life that defies the desire for consistency^1^. People deal with evaluative conflicts on a daily basis in numerous domains^2^, such as food choice^3,4^ and romantic partners^5,6^. Many attitude objects, in contrast, elicit conflict only occasionally, including some other people^7,8^, novel consumer products^9^, and vacation planning^10^. The nature of evaluative conflict about an attitude object therefore varies across time, ranging from a single occurrence in a lifetime to daily recurring episodes.

Ample evidence has shown that the experience of ambivalence can decrease well-being^11,12^, and ambivalence induces aversive states like uncertainty and anticipated regret^13,14^. This aversiveness of ambivalence motivates people to regulate the discomfort or regain a univalent attitude^15–17^. However, despite the large temporal variation inherent to the different attitude objects employed in previous research, the motivational consequences of ambivalence have been investigated in isolation from its temporal characteristics. This is noteworthy because repeated experiences of ambivalent discomfort about an attitude object may be aversive in themselves, such that recurring ambivalence could shape the way people cope with the ambivalence.

To further our understanding of the nature of ambivalence and its motivational consequences, we investigate the extent to which individuals report ambivalence to reoccur across attitude objects and whether perceived reexperiences of ambivalence influence coping efforts. We propose that the perceived recurrence of ambivalent discomfort motivates people to engage in more effortful coping than nonrecurring ambivalence, aiming to achieve a univalent attitude and thereby avert future experiences of the ambivalence. Three studies test these assertions by examining the natural variability in perceptions of how frequently ambivalence reoccurs and the motivational roles of recurrence-induced negative affect and the desire to avert future ambivalence in more effortful coping.

## The temporal dynamics of ambivalence

Psychological states inherently fluctuate across time^18,19^. In the context of attitudinal ambivalence, prevailing theorizing suggests that metacognitive awareness of evaluative conflict (i.e., felt ambivalence) arises in situations when opposed sides of an ambivalent attitude object (i.e., potential ambivalence) are simultaneously accessible^20,21^. Even for attitude objects with relatively stable potential ambivalence, such as in the domains of food and personal goals^22,23^, an experience sampling study has shown that feelings of conflict episodically fluctuate across time^2^. In the context of school-leisure conflicts in adolescents, for another example, Kuhnle et al.^24^ revealed considerable variance in the perceived frequency of evaluative conflict depending on individual differences and environmental context. A pivotal reason for why felt ambivalence arises situationally may be decision-making^8,14,15,17,25^. A recent large-scale experience sampling study^4^ showed that potential ambivalence about a topic remained in a dormant state throughout everyday life until people had to make a conflicted decision and its potential outcomes became salient, giving rise to episodes of felt ambivalence. Given the propensity of ambivalent attitudes to engender repeated instances of felt ambivalence, this line of research indicates that felt ambivalence can chronically recur.

When ambivalent discomfort towards an attitude object occurs repeatedly over multiple intervals, individuals might appraise the frequent reexperience as aversive in itself. In the context of unpleasant thoughts and emotions more generally, repeated experiences have been associated with feelings of frustration^26,27^, annoyance about the persistence^28^, concern about future outcomes and a need to gain control over the aversive state^29^. These characteristics indicate that perceptions of recurring ambivalence involve appraisals of chronic and recent reappearances of the ambivalence, as well as an anticipation of future reexperiences. Based on this proposed recurrence-induced discomfort and the ensuing desire to forestall future experiences of the ambivalence, we argue that recurring ambivalence motivates individuals to resort to effortful coping strategies that they deem effective in averting the discomfort ambivalence is known to elicit. While potential ambivalence can impact attitude stability and choice variability^30–32^, ambivalence-induced discomfort drives the motivational consequences of felt ambivalence with the aim to alleviate the discomfort^17^. People employ a vast number of coping strategies to alleviate aversive states like felt ambivalence, which have been conceptualized as either problem- or emotion-focused^15,21,33^. Emotion-focused coping primarily aims at alleviating momentary ambivalent discomfort, such as by avoiding a conflicted decision^34,35^. Problem-focused strategies like effortful information processing attempt to restore attitudinal consistency and can thus be more effective in the long run^15,17^. This effectiveness of problem-focused coping aligns with self-regulation research showing that coping strategies dedicated to preventing an aversive state are, on average, more effective than reactive coping strategies that primarily alleviate momentary affect^36–39^.

According to the Model of Ambivalent Discomfort^21^, the choice between coping strategies entails trading off the cognitive effort involved in a strategy against accuracy goals for the outcome of an ambivalent decision. The model predicts that individuals prefer frugal coping strategies to reduce ambivalence with minimal effort, unless a desire to optimize outcomes motivates more effective but costly problem-focused coping. For example, ambivalence motivates people to seek out proattitudinal information only if they lack knowledge about a topic, presumably because high knowledge renders the perceived utility of novel information negligible^40^. This illustrates that individuals draw on contextual features of the experience of ambivalence in weighing the costs of a coping strategy against its expected effectiveness for selecting the most suitable strategy.

By the same logic, perceptions of frequently recurring ambivalence could shift the trade-off between coping strategies towards choosing actions that can avert reexperiences of the ambivalence in the long run. While thought suppression or distraction, for example, may be successful at reducing a momentary experience of ambivalence, such reactive emotion-focused actions will be ineffective in averting aversive states from reoccurring^41,42^. An inflexible preference for effortless coping can hence be maladaptive if it withholds potential improvement through more effortful problem-focused coping, such as information processing^43,44^. Selecting coping actions that meet contextual demands, in contrast, carries adaptive value^45,46^. Consequently, negative appraisals of recurring ambivalence could prompt individuals to directly address the attitudinal origins of recurring ambivalence despite the cognitive costs involved, as such problem-focused coping is instrumental in gaining univalent attitude structures^15,21,47^, thereby averting the reappearance of ambivalence in the long run. We therefore expect that perceptions of frequently recurring ambivalence are aversive and motivate individuals to resort to more effortful coping with the aim of averting the ambivalence from reoccurring.

## Overview of the current research

We conducted three studies to examine the temporal nature of attitudinal ambivalence and its motivational consequences. Perceptions of frequently recurring ambivalence should motivate people to resort to more effortful coping compared to nonrecurring ambivalence. Study 1 investigated this interaction effect of felt ambivalence with its perceived recurrence on motivating effortful coping in a cross-sectional design, while also testing for potential roles of the aversive nature of recurring ambivalence and an amplification of the effect by dispositional propensity to consider future consequences. Study 2 aimed to provide quasi-experimental evidence with high ecological validity, examining ambivalence and recurrence of preexisting attitude objects orthogonally. In doing so, we substantiated the interaction effect and further qualified the role of recurrence-induced negative affect in the consequences of recurring ambivalence. Study 3 employed a behavioral task and directly manipulated expectations about ambivalence recurring in the future. Thereby, the studies investigated our central tenet that people adapt their coping efforts to the temporal nature they attribute to ambivalent discomfort.

## Method

This research was conducted in accordance with the Declaration of Helsinki and received approval from the Ethics Review Board at the University of Amsterdam and Helmut Schmidt University Hamburg. Informed Consent was obtained from all participants. The paper reports all measures, experimental manipulations, sample size calculations, and exclusions. The data files, preregistration, and supplementary material are openly accessible at OSF: https://osf.io/skwt3/?view_only%E2%80%89=%E2%80%89453950a4494b4b23a42b909f3e4b112b.

### Study 1

We propose that the perception of frequently recurring ambivalence increases the desire to engage in effortful coping for averting future experiences of ambivalence. The first study tests whether felt ambivalence interacts with its perceived recurrence in predicting the willingness to engage in more strenuous coping efforts. Felt ambivalence should motivate more effortful coping strategies such as information processing especially at high compared to low levels of recurrence. The study employs a cross-sectional design with a diverse construct space of self-generated attitude objects aimed at providing high ecological validity to shed light on the phenomenon of recurring ambivalence.

To scrutinize the mechanisms involved in the consequences of recurring ambivalence, the study accounts for possible roles of potential ambivalence, recurrent negative thoughts, attitude importance, and dispositional future orientation. We expected that the discomfort of felt ambivalence above and beyond its underlying attitude structure (i.e., potential ambivalence) motivates coping with recurring ambivalence, as the aversive state of ambivalent discomfort is deemed to be more consequential for coping processes, even though potential ambivalence might influence coping indirectly through its effects on felt ambivalence, choice variability, or attitude strength^48^. In addition, especially people high in dispositional anticipation of future consequences consider aversive future events^49^, which could increase the motivation to avert reappearances of ambivalence. For instance, the anticipation of future regret in conflicted decisions is often fundamental to the experience and consequences of felt ambivalence^14,50^. We thus assess the Consideration of Future Consequences scale^51,52^ to explore whether recurring ambivalence is more consequential in highly future-oriented people compared to other people.

The study examines whether the motivational effect of recurring ambivalence holds even when partialling out a potential role of perceived importance of an attitude object, which may correlate with recurrence^53^ and effortful coping^54^. Furthermore, we explore whether the consequences of recurring ambivalence merely parallel those of repetitive negative thoughts^55^ or extend beyond them. While the perception of recurring ambivalence may overlap with that of recurring negativity, one could argue that it additionally elicits a stronger motivation to approach the source of the ambivalent discomfort (rather than inducing primarily distress and avoidance) by effortfully integrating both positive and negative aspects of the attitude for restoring consistency and facilitate decision-making. Taken together, we hypothesized that felt ambivalence correlates with motivation for effortful coping especially if the ambivalence recurs frequently, presumably because people appraise the recurrence as aversive; and the anticipation of future experiences of the ambivalence may amplify this interaction if it leads people to engage in efforts to avert the reappearances of ambivalent discomfort.

#### Sample

The final sample included 496 Prolific workers from the UK (*M*_age_ = 34.79, *SD* = 12.73, 57.1% with university degree, 69.2% identified as female, 29.8% male, and a total of 1.0% either non-binary or transgender. We recruited a total of 533 participants, 6.9% of whom failed at least one quality check and were thus excluded, as planned in advance, i.e., a simple attention check (“select completely disagree”), bot detection utilizing reCaptcha v3^56^, or missing or nonsensical text entries for a self-generated ambivalent topic. We aimed to recruit 532 participants with a target sample size of 478 participants, accounting for an exclusion rate of up to 10%. We determined the sample size in advance in G*Power^57^ to detect an effect of β = 0.15 with alpha = 0.05 in a multiple regression approach with more than 80% power. Given that statistical power depends on correlations between predictors in multiple regression, we provide sensitivity power estimates using the R package pwr2ppl^58^; see the OSF repository for R code): the final sample size afforded at least 80% power to detect the observed two-way and three-way interaction effects of β = 0.12 and 0.09, respectively, with alpha = 0.05.

#### Procedure

Participants consented to a study on “daily life events” and wrote down a self-generated ambivalent attitude object as in previous research [e.g., 59–61]: “We want to ask you to think of a topic you personally are ambivalent about. This could be anything – as long as you have strong but mixed thoughts and feelings about it.“ Participants reported various topics, ranging from upcoming decisions to family members, covid-19, horror movies, or meat production. They answered five measures regarding the self-generated topic and, afterward, we assessed the dispositional scale.

#### Measures

#####  Importance

 In line with previous research^62^, perceived importance of the self-generated attitude object was assessed with one item to control for potential non-target effects of variation in attitude importance by different topics: “To what extent is the topic important to you?” The response scale ranged from 1 (*not at all important*) to 7 (*very important*).

##### Potential ambivalence

 We employed two split semantic differential scales^63^ to separately assess negative and positive evaluations of the self-chosen topic: “Considering only the negative [/positive] aspects of the ambivalent topic, while ignoring the positive [/negative] aspects, how negative [/positive] are your thoughts and/or feelings regarding the topic?”, *r*(494) = − 0.32, *p* < 0.001. The response scales ranged from 1 (*not at all negative* [/*positive*] to 7 (*extremely negative* [/*positive*]. We computed potential ambivalence utilizing Thompson et al.^64^’s formula: (P + N)/2 − |P − N|. High scores reflect a high magnitude of potential ambivalence (see Table 1 for means).

##### Felt ambivalence

 The Felt Ambivalence Questionnaire^20^ measured metacognitive awareness of ambivalence about the self-generated topic. The questionnaire includes three items: “Towards the ambivalent topic I feel [/have]…” with response scales that ranged from 1 (*no conflict at all/no indecision at all/completely one-sided reactions*) to 7 (*maximum conflict/maximum indecision/completely mixed reactions*). Internal consistency was sufficient, α = 0.66, considering the small number of items and good inter-item correlation, *r*(494) = 0.47, *p* < 0.001^65^.

##### Recurrence

 A three-item scale measured the perceived extent to which experiences of ambivalence about the self-generated topic recur. In line with previous research on time perception of psychological states^26,66–69^, we assessed recalled and expected experiences of ambivalence as well as its perceived overall frequency. Two items assessed the perceived temporal distance to the ambivalence, and one item measured the overall perceived frequency: “When is the last time you felt ambivalent about the topic before right now?”, “When do you expect to feel ambivalent about the topic again?”, “How frequently do you feel ambivalent about the topic?”. The response scales ranged from 1 (*a long time ago/never/very infrequently*) to 7 (*very recently/very soon/very frequently*). Internal consistency was good, α = 0.81.

##### Motivation for effortful coping 

We devised a scale to measure whether individuals explicitly want to engage in effortful coping, in line with previous research^70^. Four items measured the motivation to engage in effortful problem-focused coping (adapted from^70–73^: “I am willing to do what I can to change my ambivalence about the topic and achieve a more one-sided attitude”, “I will take action to resolve my ambivalence about the topic”, “I am motivated to get rid of my ambivalence about this topic once and for all”, “Even if it costs me a lot of effort, I would try to resolve my ambivalence about the topic”. The response scales ranged from 1 (*completely disagree*) to 4 (*neither disagree nor agree*) to 7 (*completely agree*). Internal consistency was excellent, α = 0.92.

##### Future orientation

We employed the subscale Concern with Future Consequences from a revised two-factor solution of the Consideration of Future Consequences scale^51,52^. Given the adequacy of brief versions of this scale^49^, we selected four items (items 1, 7, 13, 14) based on their factor loadings and face validity, e.g., “When I make a decision, I think about how it might affect me in the future.” The response scales ranged from 1 (*very uncharacteristic of me*) to 7 (*very characteristic of me*). Internal consistency was sufficient, α = 0.77, and similar to the original scale (α = 0.80;^51^.

#### Results

##### Heterogeneity in recurring ambivalence

**Fig. 1:**
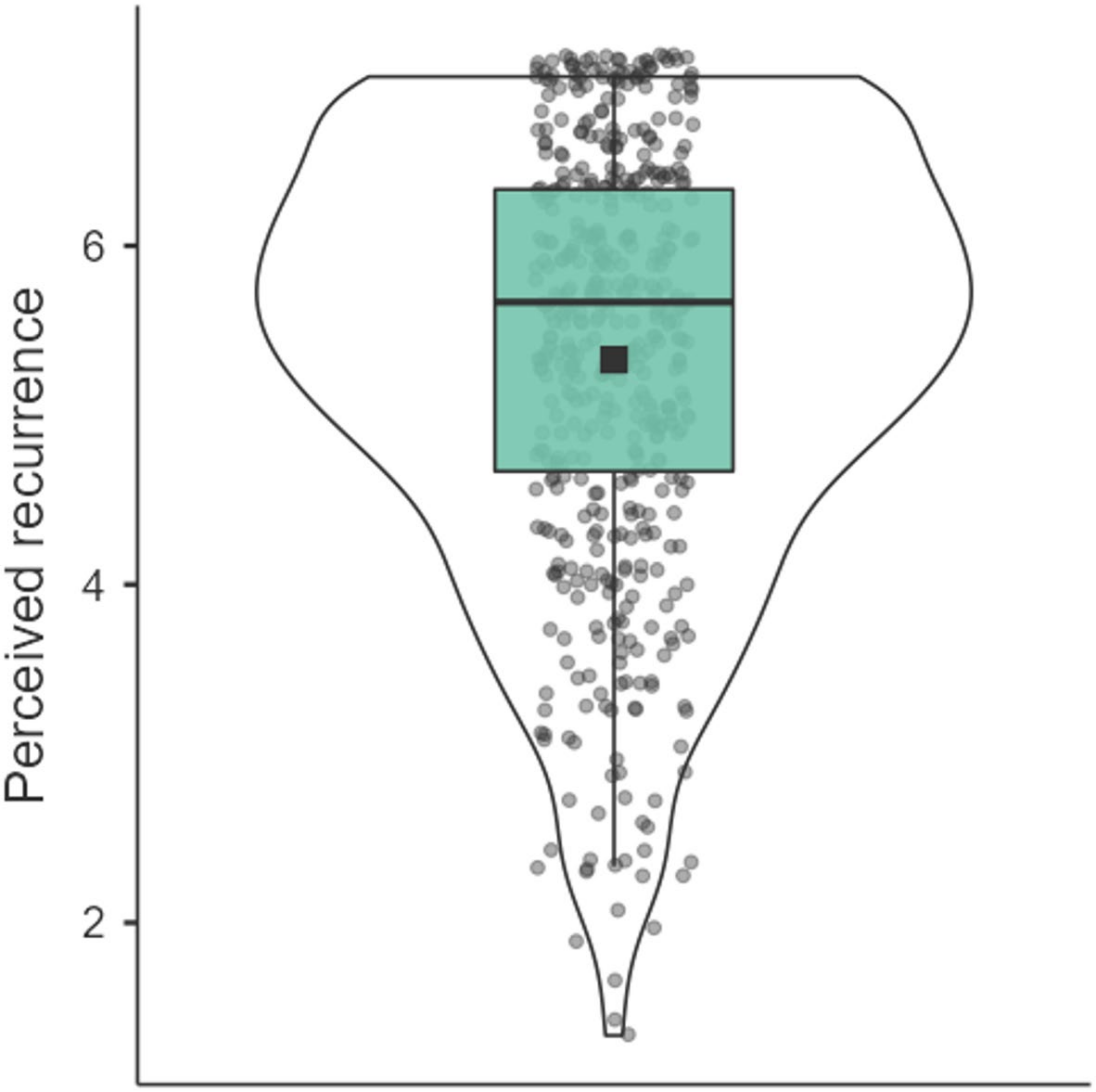
Violin plot of the variance in perceived recurrence of self-generated ambivalent attitude objects (with random jitter on the x-axis for data visibility

As expected, the reported ambivalent attitude objects varied substantially in the perceived frequency with which the ambivalence recurs, and the objects involved a high frequency of recurrence, on average (see Fig. 1; Table 1). Recurrence significantly correlated with a greater willingness to engage in effortful problem-focused coping, felt ambivalence, importance, negative evaluation, and future orientation (see Table 1). These findings highlight that ambivalence can sometimes arise repeatedly over time, rather than as a one-time occurrence. Furthermore, the perceptions of recurrence vary by features of ambivalent attitude objects and individual differences. This systematic heterogeneity aligns with our assertion that ambivalence is associated with a range of different appraisals and motivational consequences as a function of the extent to which the ambivalence recurs over time, indicating that the temporal dynamics of ambivalence could influence its motivational consequences.

**Table 1:** Summary statistics.

		M	SD	1	2	3	4		5	6	7
1	Felt ambivalence	4.53	1.13								
2	Potential ambivalence	2.86	2.06	0.21***							
3	Recurrence	5.33	1.26	0.18***	0.01						
4	Effortful coping	3.74	1.51	0.19***	− 0.05	0.20***					
5	Future orientation	5.39	0.86	0.13**	− 0.06	0.10*	0.11*				
6	Importance	5.18	1.61	0.18***	− 0.02	0.36***	0.34***		0.19***		
7	Positive evaluation	5.04	1.49	0.07	0.31^***^	− 0.01	0.07		0.04	0.17***	
8	Negative evaluation	4.69	1.59	0.08	0.53^***^	0.10^*^	− 0.05		0.01	0.00	− 0.32***

*p < .05; **p < .01; ***p < .001; *df* = 494.

##### The interaction between felt ambivalence and its recurrence

**Fig. 2:**
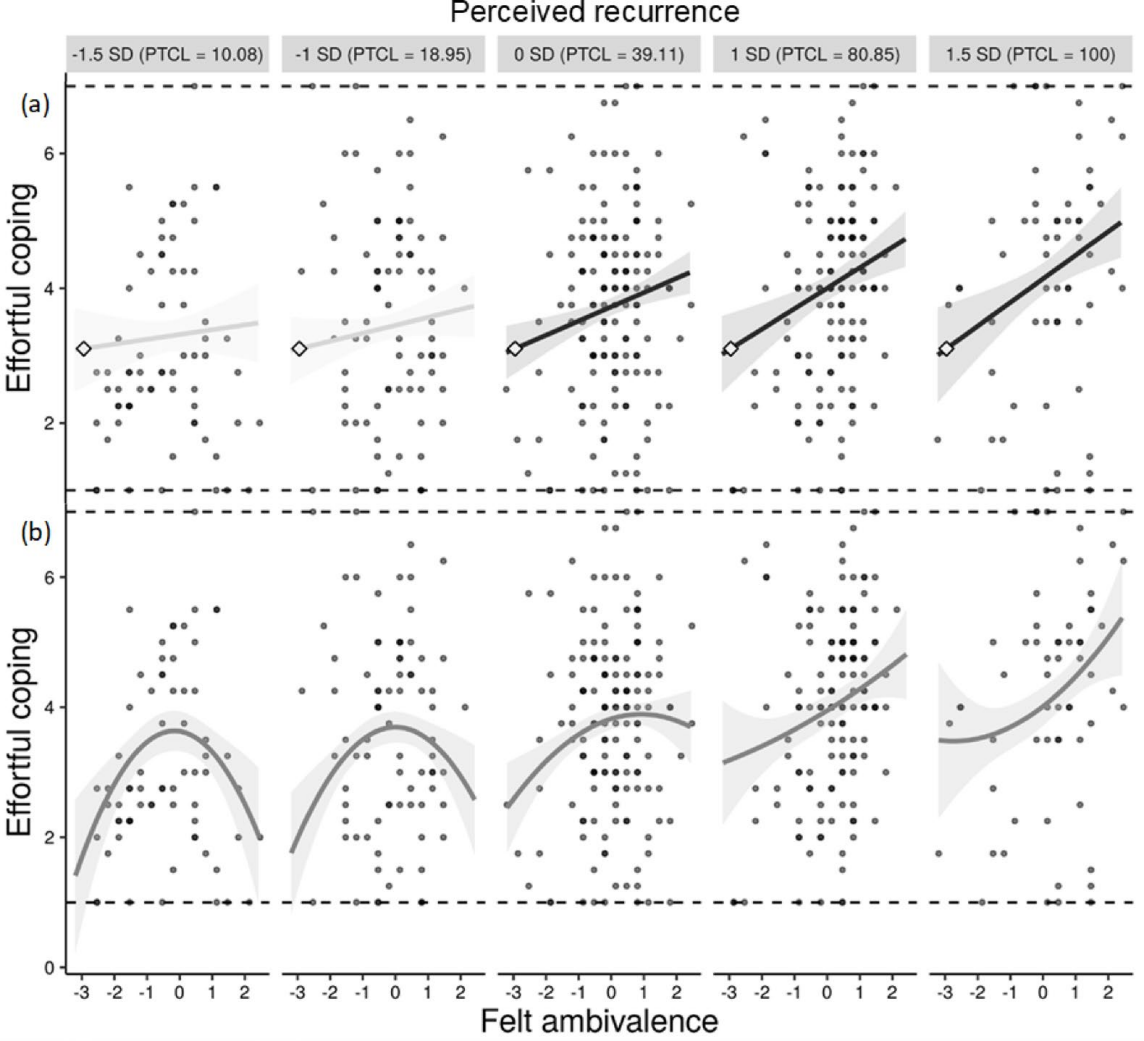
Hypothesized linear simple slopes (**a**) and non-confirmatory quadratic simple slopes (**b**) of felt ambivalence on the motivation to engage in effortful coping at five levels of perceived recurrence (using mean-centered predictor variables), with only the quadratic but not the linear effects being significant at low levels of recurrence and only the linear effects but none of the quadratic tendencies being significant at moderate and high levels of recurrence. *Note* Illustrated as recommended by McCabe et al.^80^. *PCTL* = percentiles.

We investigated an interaction effect of felt ambivalence with its perceived recurrence on the motivation to engage in effortful coping. As recommended, we conducted assumption checks to test for nonlinear effects^74–76^, which revealed a nonconfirmatory significant quadratic interaction between felt ambivalence and recurrence in addition to the expected linear effects (see Table 2; Fig. 2). This effect suggests that when recurrence is low, higher felt ambivalence promotes disengaging from effortful problem-focused coping, consistent with the idea that effortless avoidance of ambivalent discomfort is perceived as worthwhile as long as the ambivalence will not recur (see Discussion for details). We therefore report the expected linear effects beyond the nonlinear terms in accordance with best practice recommendations^74–78^ (Table S1 in the Supplementary Material reports a simple linear model, which yields the same pattern of findings). We used PROCESS^79^ to probe a model that included both linear and quadratic interaction effects^77^, providing linear (and quadratic) simple slopes while holding quadratic (or linear) effects constant^74^.

The results revealed that all main and interaction effects on the motivation for effortful coping reached significance, except for the main effect of recurrence (see Table 2). Crucially, as expected, the results for the interaction effect of a linear effect of felt ambivalence with recurrence on coping indicated that the linear simple slopes of felt ambivalence were significant at medium and high levels of recurrence, but nonsignificant at low levels (see Table 2; Fig. 2a). The Johnson-Neyman technique indicated that 60.9% of the effects were within the region of significance. These findings support our assertion that ambivalence motivates more effortful coping especially at high levels of recurrence.

In addition, the linear effects remained significant above and beyond the nonconfirmatory quadratic effects, which the model partialled out^74^. While the results of the quadratic part of the statistical model are not part of our conceptual model, they indicated a significant concave association of felt ambivalence with coping at low levels of recurrence but not at high levels (see Fig. 2b). The Johnson-Neyman technique indicated that 49.2% of the quadratic effects were within the region of significance, all of which were at low levels of recurrence. The remaining levels of recurrence, which are of focal interest to our conceptual model, are thus not characterized by quadratic effects^77^. As such, the quadratic effect of felt ambivalence at low recurrence complements the linear effects at higher levels of recurrence orthogonally to our hypotheses, indicating that recurrence impacts the pattern of ambivalence-induced coping even more so than hypothesized but in line with our reasoning. More importantly, however, the data supports our hypothesized interaction effect between ambivalence and recurrence, with higher ambivalence being related to a stronger motivation for effortful coping at higher levels of recurrence.

Next, we explored potential covariates by step-wise exclusion of nonsignificant variables (i.e., gender, age, response time), which revealed education as a significant covariate, possibly as it correlated with effortful coping, *r*(494) = − 0.21, *p* < .001, and with the interaction term between felt ambivalence and recurrence, *r*(494) = 0.09, *p* = .039. However, the pattern of findings remained the same (see Table 2, model 2).

**Table 2:** Motivation for effortful coping predicted from a linear interaction effect between felt ambivalence and perceived recurrence (accounting for the quadratic interaction term, as well as education in model 2) .

	Model 1				Model 2			
	β	95% CI		*p*	β	95% CI		*p*
		*LL*	*UL*			*LL*	*UL*	
Felt ambivalence	0.12	0.02	0.21	**0.013**	0.13	0.04	0.22	**0.004**
Felt ambivalence²	− 0.07	− 0.14	− 0.01	**0.032**	− 0.07	− 0.13	0.00	**0.039**
Recurrence	0.08	− 0.03	0.20	0.140	0.09	− 0.02	0.20	0.120
Felt ambivalence x recurrence	0.12	0.03	0.20	.**007**	0.13	0.05	0.21	.**002**
at -1 SD recurrence	0.00	− 0.13	0.13	0.993	0.01	− 0.12	0.13	0.937
at mean recurrence	0.12	0.02	0.21	**0.013**	0.13	0.04	0.22	**0.004**
at median recurrence	0.15	0.05	0.24	**0.002**	0.17	0.08	0.26	**< 0.001**
at + 1 SD recurrence	0.23	0.11	0.35	**< 0.001**	0.26	0.14	0.38	**< 0.001**
Felt ambivalence² x recurrence	0.09	0.02	0.15	**0.007**	0.08	0.02	0.14	**0.014**
at -1 SD recurrence	− 0.16	− 0.25	− 0.06	**0.001**	− 0.15	− 0.24	− 0.05	**0.003**
at mean recurrence	− 0.07	− 0.14	− 0.01	**0.032**	− 0.07	− 0.13	0.00	**0.039**
at median recurrence	− 0.05	− 0.11	0.02	0.151	− 0.05	− 0.11	0.02	0.155
at + 1 SD recurrence	0.02	− 0.07	0.10	0.720	0.01	− 0.07	0.09	0.811
Education					− 0.21	− 0.30	− 0.13	**< 0.001**
	*r*² = 0.09				*r*² = 0.13			

We report simple slopes at mean values of recurrence and additionally at the median due to a slightly skewed distribution^79^. Effect sizes are standardized.

Statistically significant p-values are highlighted in bold.

##### The motivational nature of recurring ambivalence

We hypothesized that the aversive recurrence of ambivalent discomfort motivates effortful coping. Consequently, the correlational pattern should be primarily explained by the motivational nature of felt ambivalence rather than potential ambivalence or perceived importance of an attitude object. In support of this assertion, the interaction effect between felt ambivalence and perceived recurrence on motivation for effortful coping remained significant after controlling for potential ambivalence, positivity and negativity, and attitude importance, β = 0.10, 95% CI[0.02, 0.18], *p* = .020. We further explored whether these variables interact with recurrence in predicting coping in single moderation models (see Table 3). Only potential ambivalence showed a significant interaction with recurrence, but the simple slopes were opposite to those of the interaction of felt ambivalence: the correlation between potential ambivalence and coping was not significant when recurrence was high or medium but became significant at low recurrence. This interaction term approached nonsignificance when adding the interaction between felt ambivalence and recurrence. Likewise, the latter interaction term remained significant when controlling for the interaction between recurrence and negativity. Overall, the absence of significant correlations between potential ambivalence and coping at medium and high recurrence and the aforementioned nonsignificant zero-order correlation supports the assumption that the discomfort of felt ambivalence motivates effortful coping particularly under frequent recurrence. Moreover, the absence of a role of negativity indicates that recurring ambivalence could have distinct motivational effects from repetitive negative thoughts.

Finally, we tested whether individual differences in future orientation moderated the motivational effect of recurring ambivalence on effortful coping but the three-way interaction effect was nonsignificant, β = 0.05, 95% CI[− 0.03, 0.14], *p* = .218.

**Table 3:** Key findings from seven regression models predicting effortful coping.

	Interaction term	β	95% CI	*p*
Model 1	Negativity × Recurrence	0.04	[− 0.02, 0.09]	0.175
Model 2	Positivity × Recurrence	0.00	[− 0.07, 0.06]	0.889
Model 3	Importance × Recurrence	0.03	[− 0.05, 0.11]	0.402
Model 4	Potential Ambivalence × Recurrence	0.11	[0.03, 0.20]	0.009
	at low recurrence (− 1 SD)	− 0.18	[− 0.30, − 0.05]	0.007
	at medium recurrence (M)	− 0.06	[− 0.15, 0.03]	0.167
	at high recurrence (+ 1 SD)	0.05	[− 0.06, 0.17]	0.376
Model 5	Felt Ambivalence × Recurrence	0.10	[0.01, 0.18]	0.028
	Potential Ambivalence × Recurrence	0.09	[0.00, 0.17]	0.048
Model 6	Felt Ambivalence × Recurrence	0.09	[0.01, 0.27]	0.039
	Negativity × Recurrence	0.05	[-0.06, 0.20]	0.279

#### Discussion

The findings obtained in this study support the idea that the motivational consequences of felt ambivalence for engaging in effortful coping depend on the extent to which people perceive the ambivalence to recur. At high levels of perceived recurrence, felt ambivalence correlates with a motivation for more effortful coping, presumably due to recurrence-induced discomfort and a desire to effectively avert future experiences of reappearing ambivalence. However, the present study obtained no significant evidence for dispositional future orientation qualifying the pattern of findings. Importantly, the effect of felt ambivalence held above and beyond the effect of potential ambivalence. This pattern of findings suggests that the aversive recurrence of ambivalent discomfort may play a pivotal role in recurring ambivalence leading to more effortful coping.

Interestingly, at low levels of recurrence, the effect size of the association between felt ambivalence and effortful coping was substantially lower and concave. While this quadratic effect is nonconfirmatory and secondary as it only emerged at low recurrence (whereas our theorizing concerns high recurrence), it further corroborates our theorizing and highlights the benefits of taking a temporal perspective on the consequences of ambivalence. The nonlinear effect at low levels of recurrence revealed that the motivation for effortful coping increased from low to moderate levels of infrequent episodes of felt ambivalence but plateaued at moderate levels of ambivalence and declined again when the ambivalence became higher. This pattern suggests that an isolated experience of ambivalence tends to motivate individuals to resolve the conflict only as long as the intensity of the ambivalence reaches a threshold where they withdraw from effortful coping once it becomes too intense—possibly shifting toward avoidance strategies instead of more effortful problem-focused engagement. In other words, when recurrence is low, greater ambivalence may incline individuals to disengage from the ambivalent discomfort rather than confront its source, as observed on average for the everyday attitude objects examined in this study. This finding is consistent with emotion-regulation accounts proposing that people disengage from an aversive emotional state when its intensity becomes too high before it can gather force and elicit cognitive processing; cognitive processing would be comparatively less resource-efficient during a high-intensity emotion episode as its costs increase proportionally to emotional intensity^81,82^. As such, the emotional and cognitive costs of effortful coping can exceed its comparative advantage to more frugal coping strategies that merely alleviate ambivalent discomfort^21^. Crucially, the trade-off in selecting coping strategies may shift towards effortful coping strategies that entail bearing the ambivalent discomfort for long-term benefits if the ambivalence is perceived to recur more frequently. Indeed, the data show that when ambivalence recurs more often, it is associated with effortful coping in a linear fashion, reflecting an adaptive shift in the trade-off between accuracy and effort toward addressing the root of why the ambivalence recurs.

### Study 2

Study 1 provided initial support for the notion that people adapt their coping strategy to the temporal nature of ambivalence. The study indicated that the association between felt ambivalence and effortful coping is stronger at higher levels of recurrence, which could be explained particularly by the discomfort of reexperiencing ambivalence. In Study 2, we aim to replicate and substantiate those findings using a quasi-experimental design that orthogonally compares attitude objects by ambivalence and recurrence, investigating individuals’ motivation to cope with objects high in ambivalence and recurrence as opposed to indifferent or infrequently recurring objects. Additionally, we examine the role of recurrence-induced discomfort in the motivational effect of recurring ambivalence. The metacognitive awareness of reexperiencing ambivalence could amplify negative affect about the ambivalence, eliciting a motivation to resolve the recurrence through more effortful problem-focused coping despite being cognitively taxing.

#### Sample

The final sample included 838 Prolific workers from the UK (*M*_age_ = 35.37, *SD* = 12.48, 58.0% with university degree, 75.4% identified as female, 23.9% male, 0.7% missing reports). We recruited a total of 898 participants, 6.7% of whom failed at least one quality check and were thus excluded, as planned in advance, i.e., a simple attention check (“select completely disagree”), 2.5 SD or more below the mean response time, or missing or nonsensical text entries for the attitude object. We aimed to recruit 897 participants with a target sample size of 852 participants, accounting for an exclusion rate of 5%. We determined the sample size in advance based on Study 1 to detect an interaction effect of *d* = 0.16 (β = 0.09) at *p* = 0.05 with more than 90% power in a 2 × 2 between-subjects ANOVA using Superpower^83^ as recommended by^84^. Sensitivity power analyses in G*Power^57^ indicated that the final sample size afforded 80% power to detect an effect of *d* = 0.19 and 50% power for *d* = 0.13 in the 2 × 2 ANOVA with alpha = 0.05.

#### Procedure

Participants provided informed consent and completed an *introspective ambivalence-induction task*^60,61^, which included an orthogonal manipulation of recurrence. This procedure is widely used to elicit mixed feelings while maintaining both high ecological and internal validity^59^. Participants were randomly assigned to one of four conditions in a 2 × 2 design that manipulated ambivalence (vs. indifference) and recurrence (high vs. low). In each of the conditions, a 100-word vignette explained what ambivalence (or indifference) is, while providing a temporal frame of either frequent or rare exposure. Finally, we instructed participants to write down an attitude object: “Please write down a topic you are highly [ambivalent / indifferent] about [and frequently / but rarely] think about in your [day-to-day / whole] life” (see Supplementary Material for the complete vignettes.) The subsequent questionnaire items were programmed to concern the topic each person self-generated.

#### Measures

Two items measured felt ambivalence and recurrence as manipulation checks. We utilized one item from the Felt Ambivalence Questionnaire^20^ with a response scale ranging from 1 (*no conflict at all*) to 7 (*maximum conflict*): “Towards the topic I feel…” Recurrence was measured by one question adapted from Study 1 with a response scale ranging from 1 (*very infrequently*) to 7 (*very frequently*): “How frequently do you think about the topic?”

Subsequently, we assessed the valence of affective reactions to recurrence on two items, i.e., “negative” and “positive”^85^, anchored by the question: “When you consider the frequency of you thinking about the topic, to what extent do you feel…“. The two response scales ranged from 1 (*not at all*) to 7 (*very much*). While our hypothesis only concerned negative affect, we included positive affect to counterbalance for the negative item. The two items correlated, *r*(836) = − 0.28, *p* < 0.001. Lastly, effortful problem-focused coping was assessed on the same four items as in Study 1, with excellent internal consistency, α = 0.92.

#### Results

##### Manipulation checks

**Fig. 3:**
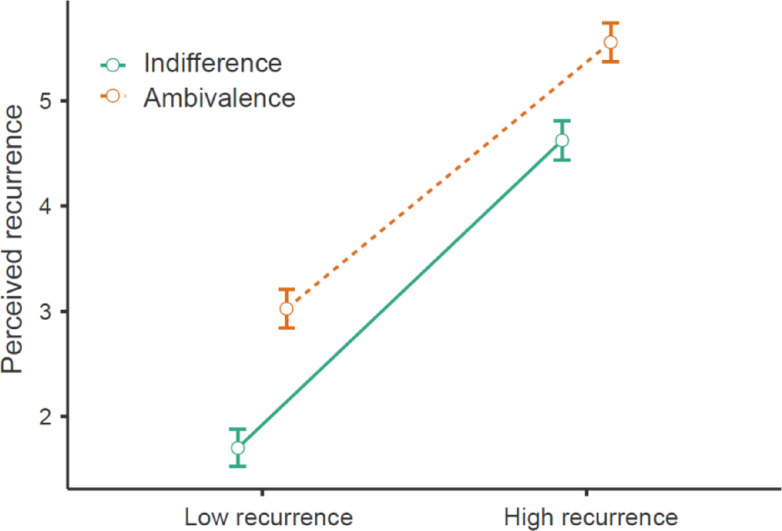
Illustration of an exploratory manipulation check of an interaction effect between the recurrence and ambivalence manipulations on perceptions of recurrence with 95% confidence intervals.

Manipulation checks (see Tables 4 and 5) replicated previous research (e.g.,^60,61^) in a two-way ANOVA by showing that felt ambivalence was substantially higher in the ambivalence condition than in the indifference condition, besides an exploratory and less pronounced main effect of recurrence. Another ANOVA showed that perceived recurrence was substantially higher in the frequent compared to the infrequent recurrence condition. Additionally, there was an exploratory main effect of ambivalence (vs. indifference) on perceived recurrence and a small crossover interaction (Fig. 3), η²_p_ = 0.01, with post-hoc tests indicating that the effect of recurrence induction within the indifference condition, *t*(834) = 22.24, *p* < .001, *d* = 2.16, was somewhat larger, *M*_diff_ = 0.39, *SE* = 0.13, *d*_*diff*_ = 0.28, than within the ambivalence condition *t*(834) = 19.11, *p* < .001, *d* = 1.88. This small difference might indicate that thinking about the ambivalent topic overshadowed by the recurrence manipulation (as in Study 3). Crucially, the direction of this interaction effect is opposite to our hypothesized effect on effortful coping. Overall, these manipulation checks suggest that the experimental conditions differed in felt ambivalence and perceived recurrence as expected, although they additionally involved unexpected effects that were less pronounced but indicate incomplete experimental control.

**Table 4:** Results from two two-way ANOVAs and post-hoc tests predicting (a) felt ambivalence and (b) perceived recurrence from the experimental manipulations.

	*p*	F(1, 834)	η²_p_
(a) Felt ambivalence			
Ambivalence manipulation	< 0.001	501.60	0.38
Recurrence manipulation	< 0.001	33.34	0.04
Ambivalence × Recurrence	0.103	2.66	0.00
(b) Perceived recurrence			
Recurrence manipulation	< 0.001	853.97	0.51
Ambivalence manipulation	< 0.001	146.11	0.15
Recurrence × Ambivalence	0.037	4.36	0.01

**Table:** Descriptives by experimental condition.

Condition		Felt ambivalence		Recurrence		Effortful coping		Negativity	
	*n*	*M*	*SD*	*M*	*SD*	*M*	*SD*	*M*	*SD*
Frequent ambivalence	210	4.92	1.40	5.56	1.21	4.44	1.39	4.71	1.51
Infrequent ambivalence	205	4.50	2.24	3.02	1.67	3.65	1.00	4.45	1.52
Frequent indifference	202	2.81	1.53	4.62	1.42	3.23	1.49	3.69	1.43
Infrequent indifference	221	2.06	1.45	1.70	1.06	2.83	1.36	3.20	1.57
Total	838	3.56	1.89	3.70	2.01	3.53	1.53	4.00	1.62

##### Main findings

**Fig. 4:**
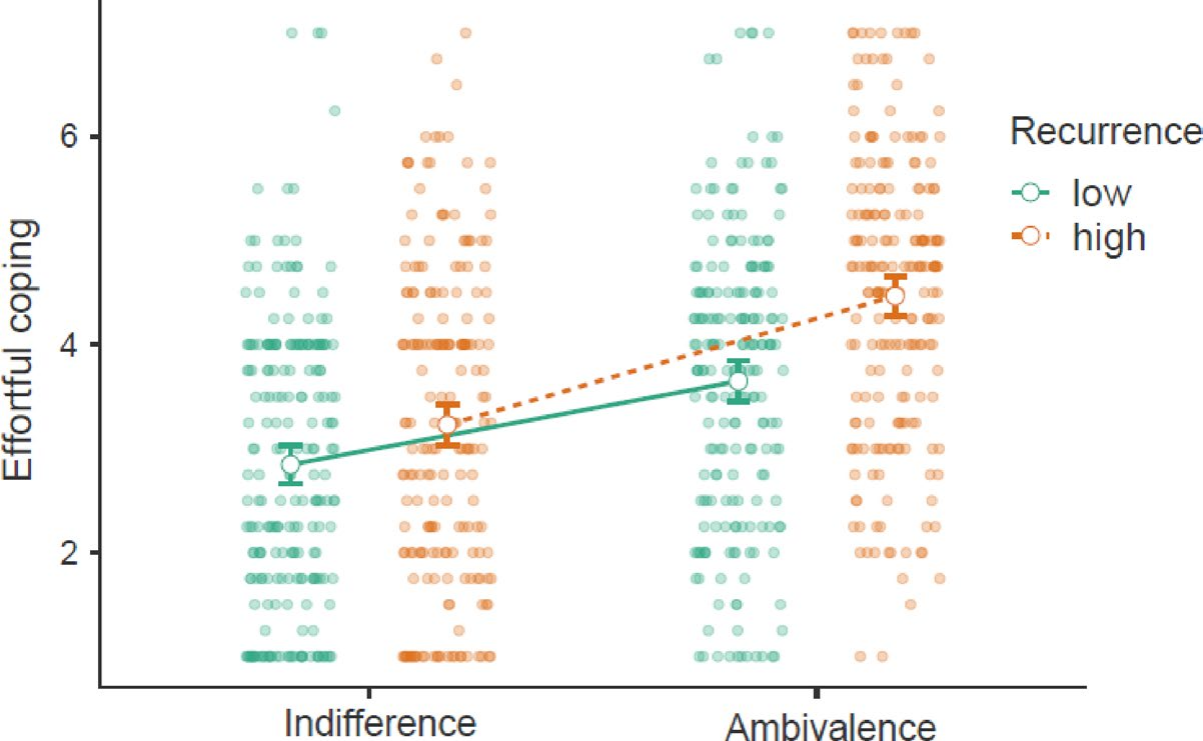
Motivation for effortful coping by ambivalence (vs. indifference) and recurrence (high vs. low) with 95% confidence intervals.

A two-way ANOVA supported our main assertion of an interaction effect between ambivalence and recurrence on motivation for effortful coping (see Fig. 4; Table 6). As hypothesized, a simple effects analysis showed greater effortful coping in the ambivalence condition with high recurrence compared to low recurrence. Next, we added potential covariates to the model. As in Study 1, education and age were significant covariates without differing by group, and the findings remained the same after controlling for them (see Table 6).

**Table 6:** Effortful coping predicted from ambivalence (vs. indifference) and recurrence (vs. nonrecurrence) in a two-way ANOVA, including simple effects of ambivalence at levels of recurrence.

	Model 1			Model 2		
	d	F(1,834) / t(834)	*p*	d	F(1,823) / t(823)	*p*
Felt ambivalence	0.71	109.05	< 0.001	0.72	120.48	< 0.001
Recurrence	0.40	37.17	< 0.001	0.40	37.84	< 0.001
Felt ambivalence x recurrence	0.13	4.06	0.044	0.14	4.94	0.027
At high recurrence	0.86	*t =* 8.74	< 0.001	0.92	*t =* 9.27	< 0.001
At low recurrence	0.58	*t =* 6.01	< 0.001	0.61	*t =* 6.24	< 0.001
Education				0.20	10.80	< 0.001
Age				0.29	22.33	< 0.001

Education and age did not significantly differ by group, *F*(3,834) = 0.52, *p* = .806, partial *η*² < 0.01, and *F*(3,834) = 1.42, *p* = .236, partial *η*² = 0.01, respectively.

##### Recurrence-induced negative affect

We expected that recurring ambivalence motivates engagement in effortful coping due to the aversive nature of the recurrence. In line with this model, a two-way ANOVA indicated that recurrence-induced negative affect was higher in the frequent compared to the infrequent recurrence condition, *F*(1,834) = 113.25, *p* < .001, *d* = 0.24, just as in the ambivalence compared to the indifference condition, *F*(1,834) = 118.43, *p* < .001, *d* = 0.75. However, opposed to our prediction, the interaction effect between ambivalence and recurrence on negative affect was nonsignificant, *F*(1,834) = 1.20, *p =* .274, *d* = 0.07. We scrutinized this finding in exploratory post-hoc comparisons, revealing that the nonsignificant interaction effect is due to unexpectedly higher negativity in the recurring indifference compared to the nonrecurring indifference condition, *t*(421) = 3.36, *p*(Bonferroni-corrected) = 0.005, *d* = 0.33 (see Table 5 for descriptives). This exploratory analysis indicates that when people frequently feel indifferent about a topic, they might appraise the recurrence as negative. For example, chronic indifference could be associated with boredom, or indifference in daily food choices might hamper decision-making and become mentally taxing when individuals cannot engage in effective decision-making due to lacking preferences. Overall, the unequivocal main effects of ambivalence and recurrence on negativity indicate that individuals generally perceive the recurrence of ambivalence as unpleasant. Given that our measure of negative affect about recurrence might be limited, such as by relying on a single-item scale of a generalized state, our next analysis offers additional insights by examining whether negative affect is systematically associated with the effect of recurring ambivalence on effortful coping.

**Fig. 5:**
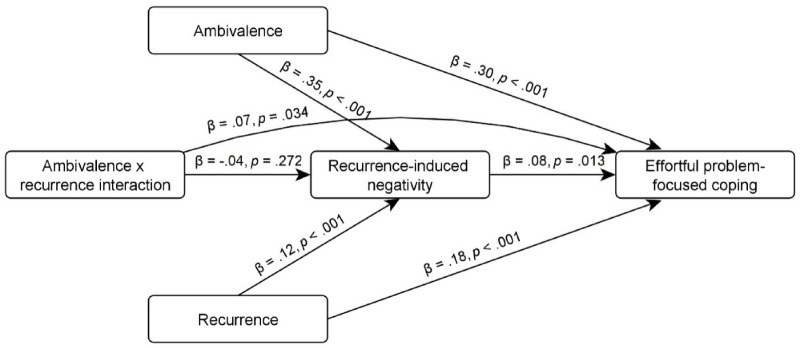
Mediation model of the consequences of ambivalence (vs. indifference), recurrence (vs. nonrecurrence), and their interaction for motivating effortful coping through recurrence-induced negative affect.* Note* The indirect effect of the interaction term was nonsignificant, β > − 0.01, *p* = .316, in contrast to the indirect effects of recurrence, β = 0.01, *p* = .041, and ambivalence, β = 0.03, *p* = .016, on coping through recurrence-induced negativity.

We conducted a mediation analysis to test for indirect effects of ambivalence, recurrence, and their interaction effect on effortful coping through recurrence-induced negative affect (see Fig. 5). All indirect and direct effects of recurrence and ambivalence were significant (except for the abovementioned indirect effect of the interaction term on negativity). Most importantly, there was a significant direct effect of negativity on coping, as well as indirect effects of ambivalence and recurrence on coping through negativity.

#### Discussion

These findings suggest that the motivation to engage in more effortful coping is associated particularly with ambivalence that is perceived as frequently recurring. Overall, the quasi-experimental data provide tentative support for the presumed role of negative affective reactions to recurring ambivalence in the motivation for effortful coping, despite a mixed pattern of findings.

While the study design tested our theorizing across a wide range of self-generated and thus natural attitude objects, this approach yields limited causal inference. The ease with which individuals can retrieve their experiences and generate topics could vary across conditions, such that when an ambivalent issue is infrequently encountered, it may be less accessible in memory. While Study 1 accounted for potential confounding variables like attitude importance, other variables like vividness of ambivalence^86^ remain unaddressed. Study 3 aimed to further address this limitation by employing decision-making task for converging evidence, which additionally provided a direct test of the role of future-oriented thinking.

### Study 3

The previous studies provided support for our assumption that individuals cope more effortfully with ambivalence if it frequently recurs. While these studies employed a broad set of naturally occurring attitudes, such ecological validity can potentially limit experimental control. Study 3 therefore examines the motivational nature of recurring ambivalence using a behavioral task in which participants judge a target person described with either positive, negative, or ambivalent information, while manipulating expectations of future recurrence.

Besides aiming to increase experimental control, the study adds to the previous studies in three ways. First, it manipulates anticipation of recurrence in the future for a direct test of the theorized role of motivation to prevent ambivalence from recurring in the future, given the nonsignificant role of dispositional future-oriented thinking in Study 1. Second, it compares recurring ambivalence to conditions with positive or negative attitudes, aiming to further disentangle a potential overlap between repetitive negative thoughts and recurring ambivalence. In contrast to unequivocal action tendencies in recurrent decision-making about an (un)favorable attitude, we propose that ambivalence can amplify the motivation to approach the attitudinal inconsistency and come to an optimal decision when individuals encounter an ambivalent decision more frequently. Study 3 thereby examines whether recurring ambivalence can elicit unique consequences or reflects a form of recurrent negative thought. Third, rather than merely measuring self-reported coping motivation as an outcome as in the previous studies, the current design allows for assessing information selection to observe the behavioral component of coping motivation.

#### Sample

The final sample included 338 Prolific workers from the UK (*M*_age_ = 39.2, *SD* = 14.0, 69.2% with university degree, 48.5% identified as female, 49.4% male, 1.8% neither). Based on previous research with a similar ambivalence induction^50^, we preregistered a required sample size of 202 participants to detect an interaction *f* = 0.29 with 90% power at *p* < 0.05 in a 3 × 2 ANOVA according to G*Power^57^.

As preregistered, we recruited 350 participants to be on the safe side and excluded eight because of failed attention check items (e.g., “select completely disagree”). Four participants were excluded for being slower than our preregistered criterion (2.5 SD above the mean). A total of 369 people started the study, 19 of which dropped out before completing it.

#### Procedure

After providing informed consent and sociodemographic data, participants were randomly assigned to one of six conditions in a 3 × 2 design, presenting information about a target person that varied in valence (ambivalent, positive, or negative) and anticipated recurrence (frequent or infrequent).

The vignettes (see preregistration) instructed participants to clean our subject pool by deciding whether a fellow Prolific worker should be excluded based on work performance. We adapted the vignettes^50^ and added an orthogonal manipulation of anticipation of future recurrence in two sentences at the end of a page with a general introduction to the task: Participants in the low recurrence condition were told they would be invited to repeat the cleaning task next year, whereas the high recurrence condition involved “such tasks continuously over the coming weeks and months”.

Subsequently, participants received information about a target person’s work performance. The ambivalence condition included three positive and three negative behaviors, whereas the other conditions reported four positive or negative behaviors and two slightly opposite behaviors (e.g., “[Correctly answered the / Failed to answer the / Failed 2 out of 3] awareness question[s]”). Participants next read about the consequences of their decision, answered the questionnaire, and eventually made a decision.

#### Measures

*Manipulation Checks*. Two split semantic differential items measured positive and negative evaluations of the target person as in Study 1, with a nonsignificant correlation within the ambivalence condition, *r*(111) = − 0.15, *p* = .118, but significant correlations within the positive, *r*(112) = − 0.41, *p* < .001, and negative conditions, *r*(115) = − 0.28, *p* = .002. Perceived recurrence was measured by one question with a response scale ranging from 1 (*never again*) to 7 (*very frequently*): “How often do you expect to continue judging similar participants in the future?”. Felt ambivalence was measured as in Study 2.

*Recurrence-induced negativity*. We adapted the measure from Study 2 by assessing affective reactions to recurrence on five items (“negative”, “annoyed”, “frustrated”, “concerned”, “uncomfortable”) anchored by the question: “When thinking about having to repeat judging similar participants in the future, to what extent do you feel each of the following?“. The response scales ranged from 1 (*not at all*) to 7 (*very much*) and were aggregated (α = .86).

*Effortful coping*. An information selection task measured effortful coping. In the first part, participants could choose additional pieces of information about the target person’s performance to receive a tailored information packet. They were instructed to choose as much or little as they wanted, requiring them to tradeoff accuracy and effort in deciding about the target person. The instructions included the question “For each item below, how much additional information would you like?” and five items (e.g., “Exact wording of the open-ended questions”) with response scales ranging from 0 (*No additional information needed*) to 10 (*All additional information*). Additionally, generalized coping effort was measured on the next page: “What is your preferred length for the information packet?” after telling participants that they could receive the selected information only via a Prolific message due to time constraints (see preregistration for the instructions). The response slider ranged from 0 (*No additional information needed*) to 10 (*10 min of information*). Internal consistency of the six items was good, α = 0.83.

#### Results

##### Manipulation checks

 We tested whether the experimental conditions manipulated felt ambivalence and perceived recurrence in four ANOVAs (see Table 7 for descriptives and Tables S2 and S3 for statistics). First, there was a main effect of valence on felt ambivalence, η²_p_ = 0.28, such that it was higher in the ambivalence condition compared to the negative and positive conditions, *d*s > 0.87.

**Fig. 6:**
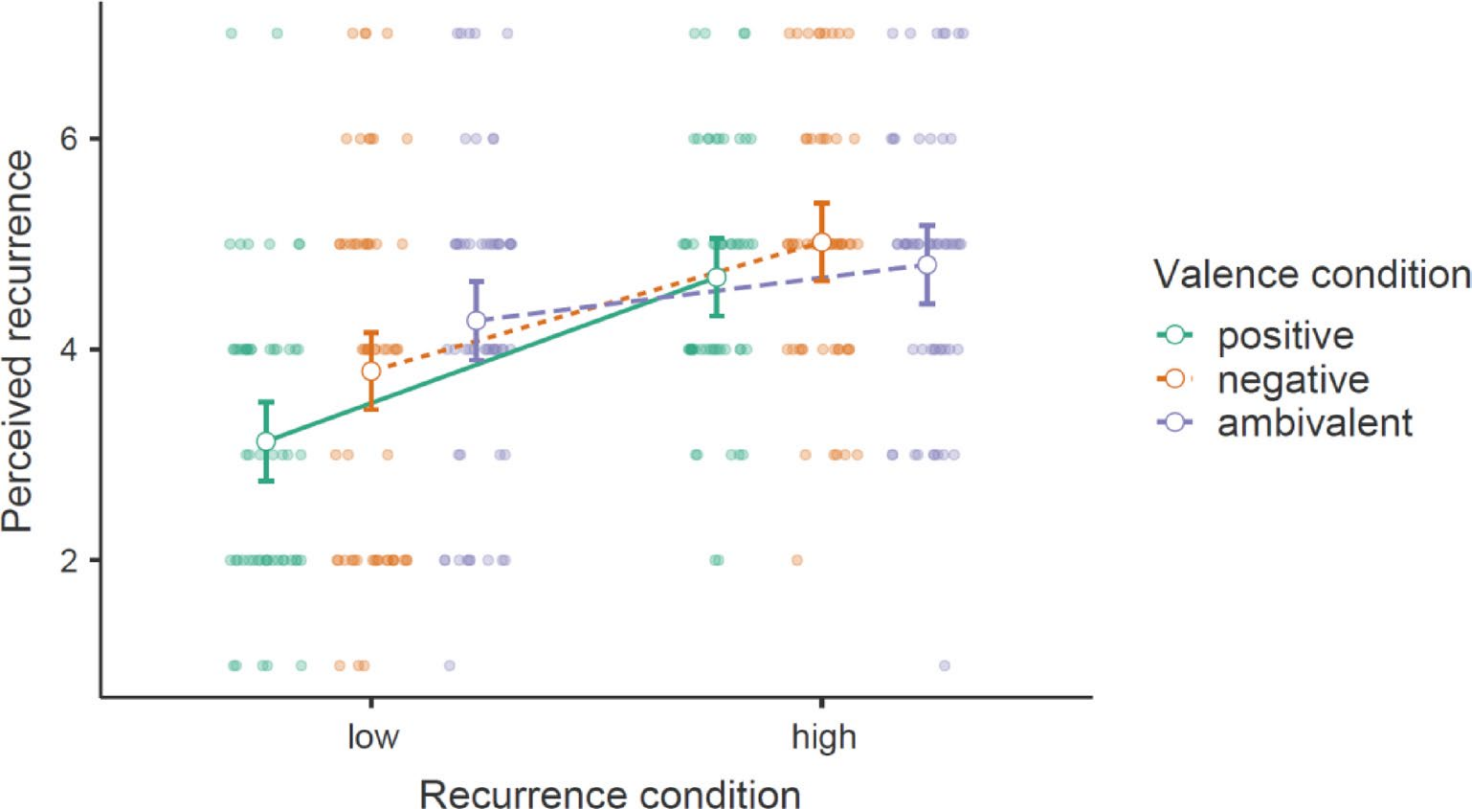
Effect of the recurrence manipulation on perceived recurrence as a function of valence, with 95% confidence intervals.

Second, perceived recurrence was higher in the frequent compared to the infrequent recurrence condition, η²_p_ = 0.13, but as in Study 2, there were significant effects of the valence manipulation and the interaction between both (see Fig. 6), such that the simple effect of the recurrence manipulation only narrowly reached statistical significance within the ambivalence condition, *p* **=** .049, *d* = 0.34, whereas the simple effects were large in the positive and negative conditions, *d*s > 0.79. The weak effect of the key experimental manipulation indicates that the ambivalence task may have overshadowed the preceding and much shorter recurrence manipulation; the direction of this effect is opposite to our hypothesized effect on effortful coping.

Third, the target persons were evaluated predominantly negatively and positively in the conditions with negative and positive targets, respectively, whereas the ambivalent target received intermediate ratings, η²_p_ > 0.53. In line with the induced valence, the behavioral choices about the target persons were predominantly favorable in the positive condition, unfavorable in the negative condition, and mixed in the ambivalent condition.

**Table 7:** Descriptive statistics for the dependent variables and manipulation checks by valence and recurrence conditions.

Valence condition	Positive		Negative		Ambivalent	
Recurrence condition	Low (*n* = 55)	High (*n* = 57)	Low (*n* = 58)	High (*n* = 57)	Low (*n* = 55)	High (*n* = 56)
Effortful coping	6.64 (2.49)	6.63 (2.43)	8.22 (2.21)	8.06 (2.14)	7.73 (1.82)	8.24 (2.25)
Negative affect	2.05 (1.49)	1.59 (0.84)	1.79 (1.04)	1.90 (1.04)	1.92 (1.12)	2.07 (0.92)
Perceived recurrence	3.13 (1.43)	4.68 (1.18)	3.79 (1.73)	5.02 (1.23)	4.27 (1.51)	4.80 (1.31)
Felt ambivalence	1.73 (0.95)	1.75 (0.95)	2.67 (1.53)	2.63 (1.62)	3.76 (1.64)	4.02 (1.66)
Negative evaluation	2.53 (1.37)	2.30 (1.12)	5.48 (1.33)	5.72 (1.25)	4.91 (1.32)	5.14 (1.03)
Positive evaluation	5.95 (0.85)	5.93 (0.90)	2.97 (1.08)	2.88 (1.32)	4.38 (1.27)	4.64 (1.45)
Choice (0 = exclude)	0.93 (0.26)	0.95 (0.23)	0.09 (0.28)	0.12 (0.33)	0.47 (0.50)	0.46 (0.50)

##### Experimental effects

**Fig. 7:**
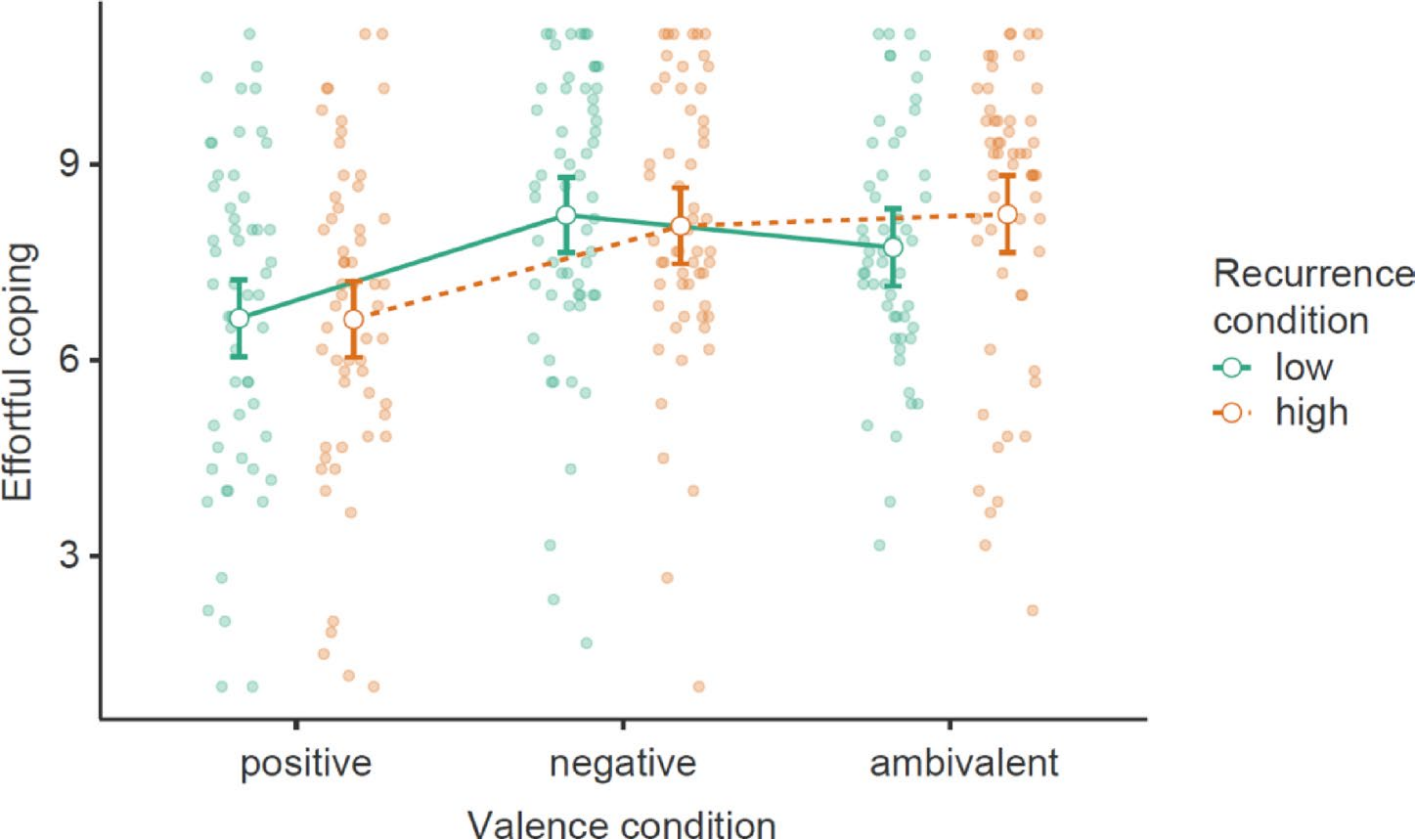
Effect of valence on effortful coping as a function of recurrence, with 95% confidence intervals

**Fig. 8:**
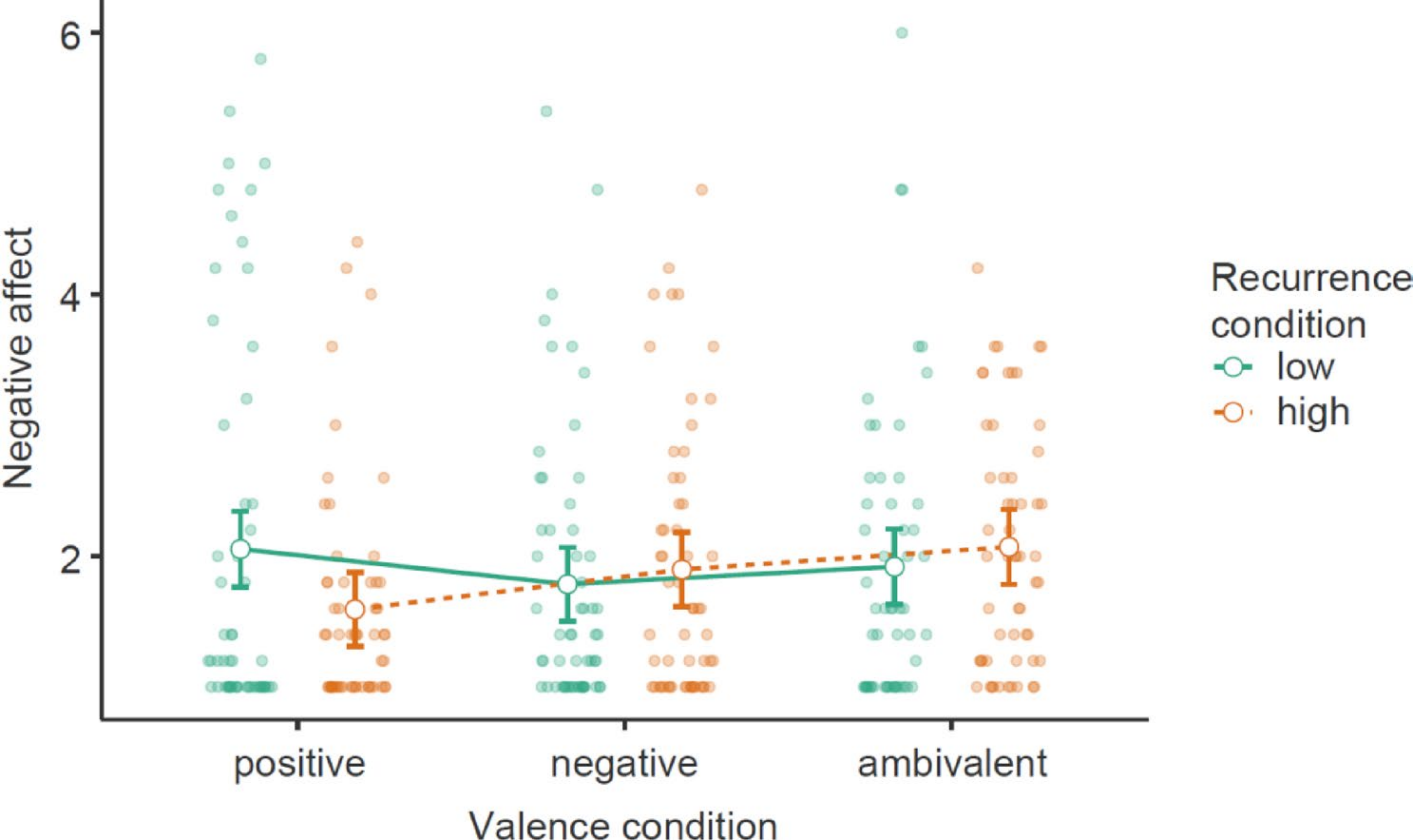
Effect of valence on recurrence-induced negative affect as a function of recurrence, with 95% confidence intervals.

We tested whether the valence and recurrence manipulations interacted in yielding higher effortful coping and recurrence-induced negative affect in two ANOVAs (see Table 8). Contrary to our predictions, there was no significant interaction effect on effortful coping, even though there was a nonsignificant but descriptive trend towards more coping in the high (vs. low) recurrence condition only within the ambivalence condition, *M*_Diff_ = 0.51 (*SE* = 0.42), *t*(332) = 1.20, *p* = .229, *d* = 0.23, but not for the other targets (see Fig. 7) The main effect of recurrence was also nonsignificant. A second ANOVA showed that the interaction effect on negative affect approached significance; however, this trend was driven by an unexpected effect within the positive condition (see Fig. 8). Given the failed experimental effects on both recurrence-induced negative affect and effortful coping, we spared running a mediation model on this predicted pathway.

**Table 8:** Results from two two-way ANOVAs predicting (a) effortful coping and (b) recurrence-induced negative affect from the experimental manipulations.

DV	Effect	F	*p*	η²*p*
Effortful coping	Valence condition	*F*(2, 332) = 15.46	< 0.001	0.09
	Recurrence condition	*F*(1, 332) = 0.20	0.653	0.00
	Valence × Recurrence	*F*(2, 332) = 0.71	0.492	0.00
Negative affect	Valence condition	*F(2*,* 332) = 0.83*	0.435	0.00
	Recurrence condition	*F(1*,* 332) = 0.31*	0.579	0.00
	Valence × Recurrence	*F(2*,* 332) = 2.77*	0.064	0.02

#### Discussion

The present study failed to observe an effect of recurring ambivalence on effortful coping. These findings were limited, first, by a small experimental effect of the recurrence manipulation within the ambivalence condition and therefore, second, insufficient statistical power. Another reason may be the absence of an experimental effect on recurrence-induced negative affect, suggesting that the induced expectation of recurring ambivalence was insufficiently undesirable. To the extent to which one can draw conclusions from those nonsignificant findings, they could indicate that merely anticipating an upcoming recurrence of ambivalence in the future (without preceding personal experiences with recurrence as in Studies 1 and 2) hardly induces aversive feelings about the recurrence and, thus, fails to motivate more effortful coping, which the General Discussion addresses further.

## General discussion

Ambivalent evaluations are ubiquitous throughout life. While previous research has investigated the experience of ambivalence and its consequences by focusing on single episodes of the ambivalence without accounting for its persistence over time, this paper sheds light on the extent to which individuals perceive felt ambivalence to naturally *reoccur* and how this perception shapes the way in which individuals cope with the ambivalence. Felt ambivalence may motivate individuals to engage in more effortful coping especially if they recurringly experience the ambivalence, presumably because of two mechanisms: recurring ambivalence could elicit recurrence-induced negative affect, and anticipating the aversive recurrence could additionally elicit a desire to more effectively resolve the ambivalence and avert future reexperiences.

Study 1 found that recurring ambivalence about an attitude object amplified the correlation of felt ambivalence with effortful problem-focused coping. Moreover, the study reveals the temporal nature of ambivalence in human experiences by highlighting considerable variability in the perceived frequency with which ambivalence about self-generated attitude objects recurs, stemming from attitude importance and individual differences in future-oriented thinking. Study 2 replicated and substantiated the interaction effect of ambivalence with its recurrence by examining how people react to recurring ambivalence as compared to indifferent or infrequent topics. We found that recurrence and ambivalence are associated with increased negative appraisals and, in turn, the motivation to engage in effortful coping. Contrary to our predictions, however, Study 3 showed no significant interaction effects between ambivalence and anticipated recurrence on negative affect and effortful coping in a behavioral task. The latter finding aligns with Study 1, which failed to observe our predicted moderation of the effect of recurring ambivalence by future-oriented thinking.

The set of studies combines high ecological validity for investigating recurring ambivalence across a broad construct space in Study 1 and 2 with Study 3’s experimental isolation of anticipated frequency of ambivalence in the future, zooming in on a subfacet of recurrence perceptions. This combination of complementary strengths may tentatively shed light on the mechanisms involved in the consequences of recurring ambivalence: Merely anticipating future instances of ambivalence based on descriptive learning may be insufficient to evoke the aversive perception of experiencing recurring ambivalence; instead, the motivational effect of recurring ambivalence could be driven primarily by the negative affect stemming from preceding experiences with the recurrence. Specifically, experiential learning about recurring ambivalence may involve an understanding of previous coping efforts being insufficient for resolving the ambivalence, which is absent in descriptive learning about future recurrence. Yet an alternative implication is that recurring ambivalence could motivate people primarily to resolve the inconsistency for the ultimate goal of averting any reexperience of the felt ambivalence (i.e., preventive coping), whereas the information task in Study 3 provided participants only with an opportunity to learn how to resolve ambivalence more successfully once they will reexperience it (i.e., reactive coping).

These potential boundary conditions, together with our mixed findings, highlight the need to isolate the effects of recurring ambivalence within a more controlled experimental design and to (a) compare which facets of recurrence underlie its motivational impact, and (b) examine how recurring ambivalence influences the selection of distinct coping strategies. Notably, alongside our interpretation that ambivalence motivates coping more strongly under high recurrence, one could also consider a complementary perspective to the extent to which higher recurrence predicts coping responses particularly at high levels of ambivalence. This may imply that the two constructs mutually amplify one another in jointly shaping regulatory responses.

Taken together, the present work contributes to prevailing theorizing on the consequences of attitudinal ambivalence^15,17,87^ by proposing that temporal dynamics in the experience of ambivalence can motivate people to avert the recurrence of felt ambivalence. In line with the notion that individuals trade off cognitive effort against accuracy goals in selecting how they cope with ambivalence^21^, individuals may adapt the choice of coping strategies to temporal features of the felt ambivalence. Specifically, we argue that individuals try to more effortfully reconcile the underlying ambivalent attitude when they experience discomfort about the persistence of ambivalence, presumably with the aim of effectively averting future reappearances of the ambivalence. Such effects of the aversiveness of recurring ambivalence may amplify the pivotal role of ambivalent discomfort in motivating coping actions^17,88^. An implication for the debate on beneficial effects of ambivalence^15,87,89–91^ is that advantageous consequences of ambivalence through effortful coping may depend on the temporal characteristics of the ambivalence, with the beneficial effects arguably being more likely to emerge when the ambivalence recurs frequently.

## Limitations and future research

The present studies are limited by the challenge of measuring generalized negative affect about recurrence. Future research could incorporate physiological measures of ambivalent discomfort or examine a more diverse set of attributions linked to possibly distinct effects of recurring ambivalence, such as anticipated relief, self-efficacy, and self-blame^92,93^. To gain further insight into the mechanisms involved in the consequences of recurring ambivalence, one could integrate negative appraisals and domain-specific concern about future outcomes^29^ to scrutinize their potential roles. Furthermore, recurring ambivalence likely motivates more effortful coping through recurrence-induced discomfort only if the ambivalence is appraised as undesirable. Thus, an experimental induction of ambivalence desirability (e.g.,^94^) should inhibit effects of recurring ambivalence.

Another worthwhile endeavor may be to investigate further boundary conditions and reduce the number of potential confounding variables. Study 1 and 2 employed the introspective ambivalence-induction task from previous research^60,61^, a widely used procedure for eliciting mixed feelings^59^. While our studies accounted for several variables, such as attitude importance, causal inference could nonetheless be limited if the self-generated attitude objects systematically differed in their cognitive accessibility, for example. A next step will therefore be to replicate our findings using different study designs, particularly by manipulating perceived recurrence within a single attitude object using repeated measurements of actual coping behavior (instead of self-reported coping motivation which limits Study 1 and 2) over time. This approach is especially well-suited for reducing limitations of our self-report measures due to social desirability bias. Because Studies 1 and 2 relied on a self-report measure of motivation for generic coping efforts, responses on these measures may involve impression-management motives. For instance, some participants possibly reported effortful coping due to a desire to signal a consistent and socially adequate response to recurring ambivalence. By the same token, such bias could have emerged both in the ambivalence and indifference condition of Study 2, or participants might have reported less effortful coping to downplay any emotional vulnerability associated with ambivalent discomfort. While the findings of Study 1 remained unchanged when controlling for attitude importance and even revealed decreased coping motivation associated with higher ambivalence at perceptions of low recurrence, a longitudinal design within a single attitude object would rigorously circumvent socially desirable responding (e.g^31,95^). Furthermore, such longitudinal data could incorporate the desire to signal consistency as a possible mechanism, besides assessing the behavioral component of effortful coping in addition to self-reported motivation.

Another question concerns whether the effects of recurring ambivalence are unique or resemble those of recurring negative attitudes, particularly repetitive negative thoughts such as rumination. While our rationale draws partly on this latter literature, the two phenomena may differ in crucial ways. Unlike unfavorable attitudes, ambivalent attitudes can evoke aversive states like feelings of conflict and uncertainty when individuals are prompted to commit to one side; these experiences can elicit motivational states that extend beyond negativity alone, as individuals strive to resolve the ambivalent discomfort and achieve a univalent stance^14,15,21^. Repeated encounters with such ambivalence may amplify these aversive states because they signal that previous attempts to resolve the conflict were ineffective, thereby evoking more effortful problem-focused coping aimed at approaching the inconsistency and enabling effective action. Unfavorable attitudes, in contrast, already afford clear action tendencies (unless they conflict with other goals), resulting in a more straightforward and cognitively less demanding coping process. Thus, although both cases can involve substantial overall negative affect and coping, individuals may engage in more effortful coping to resolve recurring ambivalence because they need to integrate both positive and negative aspects of the attitude object. While Study 2 yielded no significant interaction effect between ambivalence and recurrence on negative affect, Study 1 supported our rationale: negative evaluation played no significant roles in responses to ambivalent topics, suggesting that the observed effects are not reducible to negativity alone. Future research should further explore when the motivational consequences of recurring ambivalence and negativity diverge or overlap.

Recurring ambivalence might turn into repetitive negative thoughts when it becomes chronic over prolonged time periods as people perceive it as unresolvable and intrusive. In this case, repetitive effortful problem-focused coping may yield limited adaptiveness for averting future experiences of ambivalence. While recent research indicates that effortful coping is indeed effective in resolving ambivalence in the long run, on average^4,96,97^, such investment into an ambivalent attitude could become maladaptive in individuals who have failed to resolve ambivalence about it continuously over a longer period of time, leading them to appraise such coping as inefficient. This would eventually readjust the frequency and intensity of effortful problem-focused coping with irreconcilable ambivalence, given the pivotal role of efficacy expectations in selecting coping strategies^21^. As a consequence, individuals might possibly revert to less effortful coping for certain attitude objects at some point in time, such as by avoiding situations that make the ambivalence salient^15,90^. Moreover, the abovementioned dynamics could lead to underestimated effect sizes of recurring ambivalence in Study 1 and 2, as their designs addressed the average pattern across a large number of diverse attitude objects without accounting for the aforementioned boundary conditions that could evoke potential heterogeneous dynamics across objects and individuals.

Future research could investigate the conditions under which recurring ambivalence produces unique coping responses in several ways. For instance, experimentally manipulating a sample-based information seeking paradigm^98^ or the ease of retrieval^99^ of recurring ambivalence toward an ambivalent attitude object (compared to negative and indifferent) could more directly test for the suggested effect on increasing problem-focused rather than emotion-focused coping strategies, on average. Longitudinal designs could additionally disentangle shifts in the expected heterogeneous dynamics of these effects over prolonged time periods and examine potential nonlinear effects of recurring ambivalence as a function of dispositional coping tendencies (such as acceptance^100^ and perceived controllability in resolving the inconsistent attitude^101^. Moreover, experience sampling methods could test several interesting implications by observing the actual frequency with which ambivalence recurs in daily life^4,102^ for isolating potentially distinct roles of subjective attributions of retrospective recurrence versus (biased) affective forecasting, and for employing cross-lagged models to scrutinize whether motivational consequences of *perceived* recurring ambivalence remain significant even after accounting for momentary increases in negative evaluations and objective recurrence. Additionally, such a cross-lagged design could differentiate potentially divergent consequences of recurring ambivalence on coping through attributing the recurrence of ambivalence to poor self-efficacy as opposed to self-blame^93^.

The present work proposes that the temporal dynamics in the experience of ambivalence shape its motivational consequences in multifaceted ways. By illuminating these dynamics, a temporal perspective therefore advances our understanding of both the experience of ambivalence and the processes through which individuals cope with the ambivalence.

## Supplementary Information

Below is the link to the electronic supplementary material.

Supplementary Material 1

## Data Availability

The data files, preregistration, and supplementary material are openly accessible at https://osf.io/skwt3/?view_only=453950a4494b4b23a42b909f3e4b112b.

## References

[1] Cialdini, R. B., Trost, M. R. & Newsom, J. T. Preference for consistency: the development of a valid measure and the discovery of surprising behavioral implications. *J. Personal. Soc. Psychol.* **69**, 318 (1995).

[2] Hofmann, W., Vohs, K. D. & Baumeister, R. F. What people desire, feel conflicted About, and try to resist in everyday life. *Psychol. Sci.* **23**, 582–588 (2012). 22547657 10.1177/0956797612437426

[3] Buttlar, B. et al. The meat ambivalence questionnaire: assessing domain-specific meat-related conflict in omnivores and Vegans. *Collabra: Psychol.* https://psyarxiv.com/pnd4y/ (2023).

[4] Pauer, S., Rutjens, B. T., Hofmann, W. & van Harreveld, F. The Temporal dynamics of attitudinal conflict in daily life: An Experience sampling study of conflict emergence and resolution. (2024). https://osf.io/preprints/psyarxiv/utp46.

[5] Joel, S., Stanton, S. C. E., Page-Gould, E. & MacDonald, G. One foot out the door: Stay/Leave ambivalence predicts Day-to-Day fluctuations in commitment and intentions to end the relationship. *Eur. J. Social Psychol.*10.31219/osf.io/7gdqn (2021).

[6] Righetti, F. et al. The bittersweet taste of sacrifice: consequences for ambivalence and mixed reactions. *J. Exp. Psychol. Gen.*10.1037/xge0000750 (2020). 32150424 10.1037/xge0000750

[7] De Groeve, B. & Rosenfeld, D. L. Morally admirable or moralistically deplorable? A theoretical framework for Understanding character judgments of vegan advocates. *Appetite* **168**, 105693 (2021). 34509545 10.1016/j.appet.2021.105693

[8] Nohlen, H. U., van Harreveld, F., Rotteveel, M., Barends, A. J. & Larsen, J. T. Affective responses to ambivalence are context-dependent: A facial EMG study on the role of inconsistency and evaluative context in shaping affective responses to ambivalence. *J. Exp. Soc. Psychol.* **65**, 42–51 (2016).

[9] Jonas, K., Diehl, M. & Brömer, P. Effects of attitudinal ambivalence on information processing and Attitude-Intention consistency. *J. Exp. Soc. Psychol.* **33**, 190–210 (1997).

[10] Siddiqi, U. I. & Akhtar, N. Effects of conflicting hotel reviews shared by novice and expert traveler on attitude ambivalence: The moderating role of quality of managers’ responses. *J. Hospitality Mark. Manag.* 1–23. 10.1080/19368623.2020.1778595 (2020).

[11] Moberly, N. J. & Dickson, J. M. Goal conflict, ambivalence and psychological distress: concurrent and longitudinal relationships. *Pers. Indiv. Differ.* **129**, 38–42 (2018).

[12] Zoppolat, G., Righetti, F., Faure, R. & Schneider, I. K. A systematic study of ambivalence and well-being in romantic relationships. *Social Psychol. Personality Sci.* 194855062311655. 10.1177/19485506231165585 (2023).

[13] Nohlen, H. U., van Harreveld, F., Rotteveel, M., Lelieveld, G. J. & Crone, E. A. Evaluating ambivalence: social-cognitive and affective brain regions associated with ambivalent decision-making. *Soc. Cognit. Affect. Neurosci.* **9**, 924–931 (2014). 23685774 10.1093/scan/nst074 PMC4090963

[14] van Harreveld, F., Rutjens, B. T., Rotteveel, M., Nordgren, L. F. & Van der Pligt, J. Ambivalence and decisional conflict as a cause of psychological discomfort: feeling tense before jumping off the fence. *J. Exp. Soc. Psychol.* **45**, 167–173 (2009).

[15] Buttlar, B., Pauer, S. & van Harreveld, F. The model of ambivalent choice and dissonant commitment: an integration of dissonance and ambivalence frameworks. *Eur. Rev. Social Psychol.* **36**, 195–237 (2025).

[16] Festinger, L. *A Theory of Cognitive Dissonance* (Stanford University Press, 1957).

[17] van Harreveld, F., Nohlen, H. U. & Schneider, I. K. The ABC of Ambivalence. Affective, Behavioral, and cognitive consequences of attitudinal conflict. *Adv. Exp. Soc. Psychol.* **52**, 285–324 (2015).

[18] Bailen, N. H., Green, L. M. & Thompson, R. J. Understanding emotion in adolescents: A review of emotional Frequency, Intensity, Instability, and clarity. *Emot. Rev.* **11**, 63–73 (2019).

[19] Hurlburt, R. T. et al. Measuring the frequency of Inner-Experience characteristics. *Perspect. Psychol. Sci.* **17**, 559–571 (2022). 34283671 10.1177/1745691621990379

[20] Priester, J. R. & Petty, R. E. The gradual threshold model of ambivalence: relating the positive and negative bases of attitudes to subjective ambivalence. *J. Personal. Soc. Psychol.* **71**, 431 (1996). 10.1037//0022-3514.71.3.431 8831157

[21] van Harreveld, F., van der Pligt, J. & de Liver, Y. N. The agony of ambivalence and ways to resolve it: introducing the MAID model. *Personality Social Psychol. Rev.* **13**, 45–61 (2009). 10.1177/1088868308324518 19144904

[22] Emmons, R. A. & King, L. A. Conflict among personal strivings: Immediate and long-term implications for psychologicaland physical well-being. *J. Personality Social Psychol.* **54**, 1040–1048 (1988). 10.1037//0022-3514.54.6.1040 3397863

[23] Keller, C. & Siegrist, M. Ambivalence toward palatable food and emotional eating predict weight fluctuations. Results of a longitudinal study with four waves. *Appetite* **85**, 138–145 (2015). 25464025 10.1016/j.appet.2014.11.024

[24] Kuhnle, C., Hofer, M. & Kilian, B. The relationship of value orientations, self-control, frequency of school–leisure conflicts, and life-balance in adolescence. *Learn. Individual Differences*. **20**, 251–255 (2010).

[25] Schneider, I. K. & Schwarz, N. Mixed feelings: the case of ambivalence. *Curr. Opin. Behav. Sci.* **15**, 39–45 (2017).

[26] Linden, M., Baumann, K., Lieberei, B. & Rotter, M. The post-traumatic embitterment disorder Self-Rating scale (PTED scale). *Clin. Psychol. Psychother.* **16**, 139–147 (2009). 19229838 10.1002/cpp.610

[27] McClelland, D. C. & Apicella, F. S. A functional classification of verbal reactions to experimentally induced failure. *J. Abnorm. Social Psychol.* **40**, 376–390 (1945).

[28] Belloch, A., Morillo, C. & Giménez, A. Effects of suppressing neutral and obsession-like thoughts in normal subjects: beyond frequency. *Behav. Res. Ther.* **42**, 841–857 (2004). 15149902 10.1016/j.brat.2003.07.007

[29] Purdon, C. Appraisal of obsessional thought recurrences: impact on anxiety and mood state. *Behav. Ther.* **32**, 47–64 (2001).

[30] Armitage, C. J., Conner, M. & Attitudinal Ambivalence A test of three key hypotheses. *Pers. Soc. Psychol. Bull.* **26**, 1421–1432 (2000).

[31] Conner, M., Wilding, S., van Harreveld, F. & Dalege, J. Cognitive-Affective inconsistency and ambivalence: Impact on the overall attitude–behavior relationship. *Pers. Soc. Psychol. Bull.* 014616722094590. 10.1177/0146167220945900 (2020). 10.1177/0146167220945900 PMC7961742 32749192

[32] Luttrell, A., Petty, R. E. & Briñol, P. Ambivalence and certainty can interact to predict attitude stability over time. *J. Exp. Soc. Psychol.* **63**, 56–68 (2016).

[33] Folkman, S. & Lazarus, R. S. An analysis of coping in a Middle-Aged community sample. *J. Health Soc. Behav.* **21**, 219 (1980). 7410799

[34] Luce, M. F. Choosing to avoid: coping with negatively Emotion-Laden consumer decisions. *J. Consum. Res.* **24**, 409–433 (1998).

[35] Tversky, A. & Shafir, E. Choice under conflict: the dynamics of deferred decision. *Psychol. Sci.* **3**, 358–361 (1992).

[36] Gross, J. J. The emerging field of emotion regulation: an integrative review. *Rev. Gen. Psychol.* **2**, 271–299 (1998).

[37] Hofmann, S. G., Heering, S., Sawyer, A. T. & Asnaani, A. How to handle anxiety: the effects of reappraisal, acceptance, and suppression strategies on anxious arousal. *Behav. Res. Ther.* **47**, 389–394 (2009). 19281966 10.1016/j.brat.2009.02.010 PMC2674518

[38] John, O. P. & Gross, J. J. Healthy and unhealthy emotion regulation: personality Processes, individual Differences, and life span development. *J. Personality*. **72**, 1301–1334 (2004). 15509284 10.1111/j.1467-6494.2004.00298.x

[39] Webb, T. L., Miles, E. & Sheeran, P. Dealing with feeling: A meta-analysis of the effectiveness of strategies derived from the process model of emotion regulation. *Psychol. Bull.* **138**, 775–808 (2012). 22582737 10.1037/a0027600

[40] Sawicki, V. et al. Feeling conflicted and seeking information: when ambivalence enhances and diminishes selective exposure to attitude-consistent information. *Pers. Soc. Psychol. Bull.* **39**, 735–747 (2013). 23482502 10.1177/0146167213481388

[41] Wegner, D. M., Schneider, D. J., Iii, S. R. C. & White, T. L. Paradoxical effects of thought suppression. *J. Personality Social Psychol.* **53**, 9 (1987). 10.1037//0022-3514.53.1.5 3612492

[42] Wegner, D. M. & Gold, D. B. Fanning old flames: emotional and cognitive effects of suppressing thoughts Ofa past relationship. *J. Personality Social Psychol.* **68**, 11 (1995). 10.1037//0022-3514.68.5.782 7776182

[43] Ford, B. Q. & Troy, A. S. Reappraisal reconsidered: A closer look at the costs of an acclaimed Emotion-Regulation strategy. *Curr. Dir. Psychol. Sci.* **28**, 195–203 (2019).

[44] Mehta, A. et al. The regulation of recurrent negative emotion in the aftermath of a lost election. *Cogn. Emot.* **34**, 848–857 (2020). 31701806 10.1080/02699931.2019.1682970

[45] Cheng, C., Lau, H. P. B. & Chan, M. P. S. Coping flexibility and psychological adjustment to stressful life changes: A meta-analytic review. *Psychol. Bull.* **140**, 1582–1607 (2014). 25222637 10.1037/a0037913

[46] Lazarus, R. S. & Folkman, S. Transactional theory and research on emotions and coping. *Eur. J. Pers.* **1**, 141–169 (1987).

[47] Dalege, J., Borsboom, D., van Harreveld, F. & van der Maas, H. L. J. The attitudinal entropy (AE) framework as a general theory of individual attitudes. *Psychol. Inq.* **29**, 175–193 (2018).

[48] Conner, M. & Armitage, C. J. Attitudinal ambivalence. in Attitudes and Attitude Change. 261–286 (Psychology, New York, NY, US, (2008).

[49] Murphy, L. & Dockray, S. The consideration of future consequences and health behaviour: a meta-analysis. *Health Psychol. Rev.* **12**, 357–381 (2018). 29902949 10.1080/17437199.2018.1489298

[50] Itzchakov, G. & van Harreveld, F. Feeling torn and fearing rue: attitude ambivalence and anticipated regret as antecedents of biased information seeking. *J. Exp. Soc. Psychol.* **75**, 19–26 (2018).

[51] Joireman, J., Shaffer, M. J., Balliet, D. & Strathman, A. Promotion orientation explains why future-Oriented people exercise and eat healthy: evidence from the Two-Factor consideration of future Consequences-14 scale. *Pers. Soc. Psychol. Bull.* **38**, 1272–1287 (2012). 22833533 10.1177/0146167212449362

[52] Strathman, A., Gleicher, F., Boninger, D. S. & Edwards, C. S. The consideration of future consequences: weighing immediate and distant outcomes of behavior. *J. Personal. Soc. Psychol.* **66**, 742–752 (1994).

[53] Andrews-Hanna, J. R. et al. A Penny for your thoughts: dimensions of self-generated thought content and relationships with individual differences in emotional wellbeing. *Front Psychol* **4**, (2013). 10.3389/fpsyg.2013.00900 PMC3843223 24376427

[54] Erber, R. & Fiske, S. T. Outcome dependency and attention to inconsistent information. *J. Personality Social Psychol.* **47**, 704–726 (1984).

[55] Watkins, E. R. Constructive and unconstructive repetitive thought. *Psychol. Bull.* **134**, 163–206 (2008). 18298268 10.1037/0033-2909.134.2.163 PMC2672052

[56] Google. reCAPTCHA v3. (2022). https://developers.google.com/recaptcha/docs/v3.

[57] Faul, F., Erdfelder, E., Lang, A. G. & Buchner, A. G*Power 3: A flexible statistical power analysis program for the social, behavioral, and biomedical sciences. *Behav. Res. Methods*. **39**, 175–191 (2007). 17695343 10.3758/bf03193146

[58] Aberson, C. L. *Applied Power Analysis for the Behavioral Sciences* (Routledge, Taylor & Francis Group, 2019).

[59] Berrios, R., Totterdell, P. & Kellett, S. Eliciting mixed emotions: a meta-analysis comparing models, types, and measures. *Front Psychol* **6**, (2015). 10.3389/fpsyg.2015.00428 PMC4397957 25926805

[60] Schneider, I. K. et al. One way and the other: the bidirectional relationship between ambivalence and body movement. *Psychol. Sci.* **24**, 319–325 (2013). 23355393 10.1177/0956797612457393

[61] van Harreveld, F., Rutjens, B. T., Schneider, I. K., Nohlen, H. U. & Keskinis, K. In doubt and disorderly: ambivalence promotes compensatory perceptions of order. *J. Exp. Psychol. Gen.* **143**, 1666–1676 (2014). 24588217 10.1037/a0036099

[62] Krosnick, J. A. Attitude importance and attitude accessibility. *Pers. Soc. Psychol. Bull.* **15**, 297–308 (1989).

[63] Kaplan, K. J. On the ambivalence-indifference problem in attitude theory and measurement: A suggested modification of the semantic differential technique. *Psychol. Bull.* **77**, 361–372 (1972).

[64] Thompson, M. M., Zanna, M. P. & Griffin, D. W. Let’s not be indifferent about (attitudinal) ambivalence. *Attitude Strength: Antecedents Consequences*. **4**, 361–386 (1995).

[65] Briggs, S. R. & Cheek, J. M. The role of factor analysis in the development and evaluation of personality scales. *J. Personality*. **54**, 106–148 (1986).

[66] Diener, E., Larsen, R. J., Levine, S. & Emmons, R. A. Intensity and frequency: dimensions underlying positive and negative affect. *J. Personality Social Psychol.* **48**, 1253–1265 (1985). 10.1037//0022-3514.48.5.1253 3998989

[67] Heavey, C. L. et al. Measuring the frequency of inner-Experience characteristics by Self-Report: the Nevada inner experience questionnaire. *Front. Psychol.* **9**, 2615 (2019). 30687148 10.3389/fpsyg.2018.02615 PMC6338092

[68] Pauer, S. et al. Is the effect of trust on risk perceptions a matter of Knowledge, Control, and time? An extension and direct replication attempt of siegrist and Cvetkovich (2000). *Social Psychol. Personality Sci.* **15**, 1008–1023 (2024).

[69] Zimbardo, P. G. & Boyd, J. N. Putting time in perspective: A valid, reliable individual-differences metric. *J. Personal. Soc. Psychol.* **77**, 1271–1288 (1999).

[70] DeMarree, K. G., Wheeler, C., Briñol, S., Petty, R. E. & P. & Wanting other attitudes: Actual–desired attitude discrepancies predict feelings of ambivalence and ambivalence consequences. *J. Exp. Soc. Psychol.* **53**, 5–18 (2014).

[71] Carver, C. S., Weintraub, J. K. & Scheier, M. F. Assessing coping strategies: A theoretically based approach. *J. Personality Social Psychol.* **56**, 267–283 (1989). 10.1037//0022-3514.56.2.267 2926629

[72] Pearlin, L. I. & Schooler, C. The structure of coping. *J. Health Soc. Behav.* **19**, 2 (1978). 649936

[73] Wrosch, C., Heckhausen, J. & Lachman, M. E. Primary and secondary control strategies for managing health and financial stress across adulthood. *Psychol. Aging*. **15**, 387–399 (2000). 11014704 10.1037//0882-7974.15.3.387

[74] Cohen, J., Cohen, P., West, S. G. & Aiken, L. S. *Applied Multiple Regression/Correlation Analysis for the Behavioral Sciences* (Erlbaum Associates, 2003).

[75] Cortina, J. M. & Interaction Nonlinearity, and multicollinearity: implications for multiple regression. *J. Manag.* **19**, 915–922 (1993).

[76] Simonsohn, U. Interacting with curves: how to validly test and probe interactions in the real (Nonlinear) world. *Adv. Methods Practices Psychol. Sci.* **7**, 1–22 (2024).

[77] Hayes, A. F. & Hacking process for estimation and probing of linear moderation of quadratic effects and quadratic moderation of linear effects. *unpublished* 1–18 (2017).

[78] Roisman, G. I. et al. Distinguishing differential susceptibility from diathesis–stress: recommendations for evaluating interaction effects. *Dev. Psychopathol.* **24**, 389–409 (2012). 22559121 10.1017/S0954579412000065

[79] Hayes, A. F. *Introduction To Mediation, Moderation, and Conditional Process Analysis: A Regression-Based Approach* (The Guilford Press, 2018).

[80] McCabe, C. J., Kim, D. S. & King, K. M. Improving present practices in the visual display of interactions. *Adv. Methods Practices Psychol. Sci.* **1**, 147–165 (2018). 10.1177/2515245917746792 PMC8078831 33912789

[81] Sheppes, G., Scheibe, S., Suri, G. & Gross, J. J. Emotion-Regulation choice. *Psychol. Sci.* **22**, 1391–1396 (2011). 21960251 10.1177/0956797611418350

[82] Sheppes, G. & Gross, J. J. Is timing everything? Temporal considerations in emotion regulation. *Pers. Soc. Psychol. Rev.* **15**, 319–331 (2011). 21233326 10.1177/1088868310395778

[83] Lakens, D. & Caldwell, A. R. *Simulation-Based Power-Analysis for Factorial ANOVA Designs*. (2019). https://osf.io/baxsf

[84] Giner-Sorolla, R. et al. Power to detect what? Considerations for planning and evaluating sample size. *Personality Social Psychol. Rev.* **28**, 276–301 (2024). 10.1177/10888683241228328 PMC11193916 38345247

[85] Diener, E. et al. New Well-being measures: short scales to assess flourishing and positive and negative feelings. *Soc. Indic. Res.* **97**, 143–156 (2010).

[86] Itzchakov, G., Amar, M. & Van Harreveld, F. Don’t let the facts ruin a good story: the effect of vivid reviews on attitude ambivalence and its coping mechanisms. *J. Exp. Soc. Psychol.* **88**, 103938 (2020).

[87] Rothman, N. B., Pratt, M. G., Rees, L. & Vogus, T. J. Understanding the dual nature of ambivalence: why and when ambivalence leads to good and bad outcomes. *Acad. Manag. Ann.* **11**, 33–72 (2017).

[88] Vaccaro, A. G., Kaplan, J. T., Damasio, A. & Bittersweet The neuroscience of ambivalent affect. *Perspect. Psychol. Sci.* **15**, 1187–1199 (2020). 32758063 10.1177/1745691620927708

[89] Buttlar, B. & Pauer, S. Disentangling the Meat Paradox: A Comparative Review of Meat-Related Ambivalence and Dissonance. (2024). https://osf.io/preprints/psyarxiv/6kse4. 10.1111/bjso.70062 PMC12979709 41814466

[90] Pauer, S., Rutjens, B. T., Ruby, M. B., Perino, G. & van Harreveld, F. Meating conflict: Toward a model of ambivalence-motivated reduction of meat consumption. *Foods* **11**, 921 (2022). 35407008 10.3390/foods11070921 PMC9040712

[91] Schneider, I. K., Novin, S., Harreveld, F. & Genschow, O. Benefits of being ambivalent: the relationship between trait ambivalence and attribution biases. *Br. J. Soc. Psychol.*10.1111/bjso.12417 (2021). 10.1111/bjso.12417 32893893

[92] Sweeny, K. & Vohs, K. D. On near misses and completed tasks: the nature of relief. *Psychol. Sci.* **23**, 464–468 (2012). 22477104 10.1177/0956797611434590

[93] Kirchner, T. R., Shiffman, S. & Wileyto, E. P. Relapse dynamics during smoking cessation: recurrent abstinence violation effects and lapse-relapse progression. *J. Abnorm. Psychol.* **121**, 187–197 (2012). 21787035 10.1037/a0024451 PMC3296289

[94] Siev, J. J. & Petty, R. E. Ambivalent attitudes promote support for extreme political actions. *Sci. Adv.* **10**, eadn2965 (2024). 38865461 10.1126/sciadv.adn2965 PMC11168463

[95] Conner, M. et al. Moderator effects of attitudinal ambivalence on attitude-behaviour relationships. *Eur. J. Social Psychol.* **32**, 705–718 (2002).

[96] Buttlar, B., Pauer, S., Ruby, M. & Sherrer, V. Two sides of the same fence: A model of the origins and consequences of meat-related conflict in omnivores and vegans. *J. Environ. Psychol.* (2024).

[97] Finkhäuser, M., Sherrer, V., Pauer, S. & Buttlar, B. Feeling pushed and feeling pulled: A panel study on the temporal dynamics of meat-related ambivalence, morality, and behavioural consequences. *Soc. Psychol. Pers. Sci. *(2025).

[98] Buttlar, B., Lambrich, A., McCaughey, L. & Schneider, I. K. Too much information? A systematic investigation of the antecedents and consequences of ambivalence-induced information seeking behavior. *J. Exp. Soc. Psychol.* **121**, 104783 (2025).

[99] Schwarz, N. et al. Ease of retrieval as information: another look at the availability heuristic. *J. Personality Social Psychol.* **61**, 195–202 (1991).

[100] Kelly, R. E., Wood, A. M., Shearman, K., Phillips, S. & Mansell, W. Encouraging acceptance of ambivalence using the expressive writing paradigm: A writing intervention for ambivalence. *Psychol. Psychotherapy: Theory Res. Pract.* **85**, 220–228 (2012). 10.1111/j.2044-8341.2011.02023.x 22903911

[101] Pauer, S., Linne, R. & Erb, H. P. From the illusion of choice to actual control: reconsidering the induced compliance paradigm of cognitive dissonance. *Adv. Methods Practices Psychol. Sci.* **7**, 1–5 (2024).

[102] Buttlar, B., Pauer, S., Scherrer, V. & Hofmann, W. Attitude-based self-regulation: An experience sampling study on the role of attitudes in the experience and resolution of self-control conflicts in the context of vegetarians. *Motivation Sci.* **11**, 291–305 (2024).

