# Supplementary material for: The experience of recurring ambivalence and its relation to effortful problem-focused coping
Source: Sci Rep. 2026 Jan 16;16:2601. doi: 10.1038/s41598-026-35032-4 (PMC12820227; doi:10.1038/s41598-026-35032-4)
Supplement: Supplementary file 1 — Supplementary Material 1 [file 41598_2026_35032_MOESM1_ESM.pdf]

Supplementary Material

The experience of recurring ambivalence and its relation to effortful problem-focused coping

Shiva Pauer, Bastiaan T. Rutjens, Frenk van Harreveld

Table S1

Motivation for effortful coping predicted from a linear effect of felt ambivalence, perceived recurrence, and their interaction. Model 2 includes a covariate that was retained after step-wise exclusion of potentially confounding variables. We report simple slopes at mean values of recurrence and additionally at the median due to a skewed distribution (Hayes, 2018). Effect sizes are standardized. In accordance with best practice recommendations, the main manuscript reports the predicted linear effects beyond the quadratic interaction terms (Cohen et al., 2003; Cortina, 1993; Hayes, 2017; Roisman et al., 2012; Simonsohn, 2024).

	Model 1				Model 2			
	β	95% CI		*p*	β	95% CI		*p*
		LL	*UL*			*LL*	*UL*	
Felt ambivalence	.16	.07	.24	<.001	.17	.12	.35	<.001
Recurrence	.18	.09	.27	<.001	.17	.10	.31	<.001
Felt ambivalence * recurrence	.07	-.01	.15	.088	.09	.01	.18	.027
at -1 SD recurrence	.09	-.03	.20	.138	.08	-.04	.27	.143
at Mean recurrence	.16	.07	.24	<.001	.17	.12	.35	<.001
at Median recurrence	.18	.09	.27	<.001	.20	.11	.28	<.001
at +1 SD recurrence	.23	.11	.35	<.001	.26	.19	.51	<.001
Education					-.22	-.31	-.14	<.001
	$r^2 = .07$				$r^2 = .12$			

1. **Study 2: Experimental manipulations**
   - **High ambivalence x recurrence** [high/low]; 130 words:

     Having **both strong positive and negative** thoughts and feelings about a topic is what is known as ambivalence. Many people hold highly ambivalent attitudes about topics they [frequently / rarely] think about in their [day-to-day / whole] life, ranging from personal issues (e.g., ambivalence about a person or decision) to societal topics (e.g., ambivalence about controversial issues). We want to ask you to reflect on a topic you personally are highly ambivalent about [and frequently / but rarely] think about. This could be anything – as long as you have strong but mixed thoughts and feelings about it which you [often / infrequently] encounter.

     Please write down a topic you are highly ambivalent about [and frequently / but rarely] think about in your [day-to-day / whole] life (max. 10 words). The following questions will concern the topic you choose.

     [text entry]

- **Indifference condition** x **recurrence** [high/low], 130 words:

  Having **neither positive nor negative** thoughts and feelings about a topic is what is known as indifference. Many people hold completely indifferent attitudes about topics they [frequently / rarely] think about in their [day-to-day / whole] life, ranging from personal issues (e.g., indifference about an object or event) to societal topics (e.g., indifference about trivial issues). We want to ask you to reflect on a topic you personally are completely indifferent about [but frequently / and rarely] think about. This could be anything – as long as you have for the most part neutral thoughts and feelings about it which you [often / infrequently] encounter.

  Please write down a topic you are indifferent about [but frequently / and rarely] think about in your [day-to-day / whole] life (max. 10 words). The following questions will concern the topic you choose.

  [text entry]

Table S2

Analysis of Covariance for Manipulations Checks of the Experimental Effects on Felt Ambivalence, Perceived Recurrence, Positive Evaluation, and Negative Evaluation in Study 3

DV	Effect	*F*	*p*	η^2_p
Felt ambivalence	Valence manipulation	$F(2, 332) = 63.70$	< .001	.28
	Recurrence manipulation	$F(1, 332) = 0.27$	.606	.00
	Valence × Recurrence	$F(2, 332) = 0.33$	.720	.00
Perceived recurrence	Recurrence manipulation	$F(1, 332) = 51.59$	< .001	.13
	Valence manipulation	$F(2, 332) = 6.23$	.002	.04
	Recurrence × Valence	$F(2, 332) = 3.83$	.023	.02
Negative evaluation	Valence manipulation	$F(2, 332) = 210.60$	< .001	.56
	Recurrence manipulation	$F(1, 332) = 0.35$	.553	.00
	Valence × Recurrence	$F(2, 332) = 1.31$	.273	.01
Positive evaluation	Valence manipulation	$F(2, 332) = 190.48$	< .001	.53
	Recurrence manipulation	$F(1, 332) = 0.17$	.680	.00
	Valence × Recurrence	$F(2, 332) = 0.70$	.496	.00

Table S3

Post-Hoc Comparisons and Simple Effects for Manipulations Checks of the Experimental Effects on Felt Ambivalence, Perceived Recurrence, Positive Evaluation, and Negative Evaluation in Study 3

DV		M_{Diff}	*SE*	*t*(332)	*p*	*d*
Felt ambivalence	Post-hoc comparisons between valence conditions:					
	Positive – Negative	−0.91	0.19	−4.81	< .001	−0.64
	Positive – Ambivalent	−2.15	0.19	−11.24	< .001	−1.51
	Negative – Ambivalent	−1.24	0.19	−6.52	< .001	−0.87
Perceived recurrence	Simple effects of the recurrence manipulation within:					
	Positive condition	1.56	0.27	5.83	< .001	1.01
	Negative condition	1.22	0.26	4.65	< .001	0.79
	Ambivalence condition	0.53	0.27	1.98	.049	0.34
	Post-hoc comparisons between valence conditions:					
Negative evaluation	Positive – Negative	−3.19	0.17	−19.32	< .001	−2.56
	Positive – Ambivalent	−2.61	0.17	−15.69	< .001	−2.10
	Negative – Ambivalent	0.58	0.17	3.48	.002	0.46
Positive evaluation	Positive – Negative	3.02	0.15	19.50	< .001	2.59
	Positive – Ambivalent	1.43	0.16	9.14	< .001	1.22
	Negative – Ambivalent	−1.59	0.15	−10.26	< .001	−1.37
